# Simulating Freely Diffusing Single-Molecule FRET Data with Consideration of Protein Conformational Dynamics

**DOI:** 10.1021/acs.jpcb.5c06346

**Published:** 2026-01-15

**Authors:** James Losey, Michael Jauch, Axel Cortes-Cubero, Haoxuan Wu, Adithya Polasa, Stephanie Sauve, Roberto Rivera, David S. Matteson, Mahmoud Moradi

**Affiliations:** † Department of Chemistry and Biochemistry, 3341 University of Arkansas, Fayetteville, Arkansas 72701, United States; ‡ Department of Statistics, 7823 Florida State University, Tallahassee, Florida 32306, United States; § Department of Mathematical Sciences, 16146 University of Puerto Rico, Mayaguez, Puerto Rico 00681, United States; ∥ Department of Statistics and Data Science, 5922 Cornell University, Ithaca, New York 14850, United States

## Abstract

Single-molecule Förster resonance energy transfer (smFRET) experiments have greatly contributed to the understanding of the conformational dynamics of proteins and other biomolecules. Generating high-fidelity simulated data for smFRET experiments is an important step toward developing and examining accurate and efficient smFRET data analysis techniques. Here, we use distributions of interdye distances generated using Langevin dynamics to simulate freely diffusing smFRET timestamp data for proteins and biomolecules that have conformational flexibility. We then compare analysis techniques for smFRET data to validate the new module. The Langevin dynamics is used here as an illustrative example to demonstrate how modeling conformational dynamics can be integrated with molecular diffusion and photon emission statistics, all of which are essential for realistic simulation of freely diffusing smFRET data. We also discuss different ways to generalize our approach to make the simulated data more realistic including the employment of molecular dynamics (MD) simulations that is illustrated with an example. The Langevin dynamics module provides a framework for generating timestamp data for systems with a known underlying conformational heterogeneity as a step toward the development of new analysis techniques for smFRET data dealing with flexible proteins or other biomolecular systems.

## Graphical abstract

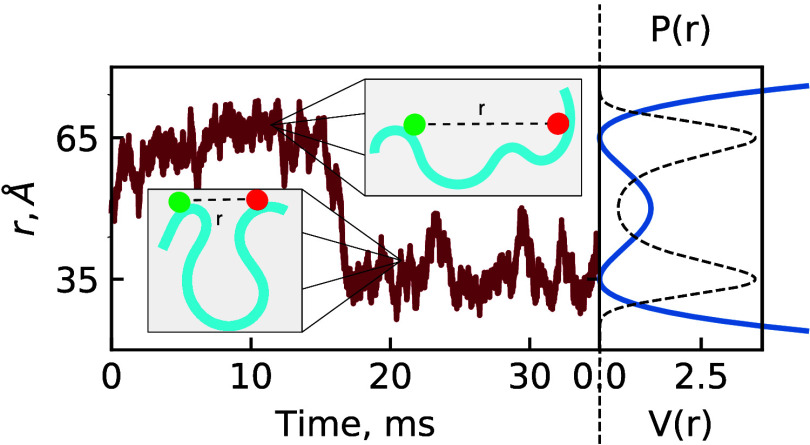

## Introduction

Freely diffusing smFRET experiments probe bimolecular dynamics on time scales where conformational heterogeneity, translational diffusion, and photon emission statistics are intrinsically coupled. Despite extensive progress in smFRET data analysis and simulation, existing simulation frameworks typically treat these components in isolation—most commonly by representing intramolecular dynamics through fixed FRET efficiencies or discrete state switching, even when the underlying conformational motion is continuous. As a result, current freely diffusing smFRET simulations lack a general mechanism for incorporating realistic conformational dynamics into detector-level timestamp data. In this work, we introduce a general framework for enriching freely diffusing smFRET simulations with physically motivated conformational dynamics. The central contribution is introducing a unified simulation strategy that allows realistic intramolecular dynamics to be propagated alongside three-dimensional diffusion and photon emission to generate realistic freely diffusing smFRET data. As a concrete and computationally efficient example, we demonstrate this approach using overdamped Langevin dynamics to evolve dye–dye distances on prescribed free-energy landscapes; however, the framework is agnostic to the specific dynamical model and naturally generalizes to higher-dimensional Langevin descriptions and to distance trajectories derived from atomistic MD simulations.

The remainder of the Introduction places this framework in the context of smFRET technique and its freely diffusing variant, outlines current freely diffusing smFRET data analysis and simulatin approaches and their limitations, and motivates the need for more realistic models of conformational dynamics and heterogeneity.

Proteins and other biomolecules rely on their structure and dynamic properties to execute their functions.^1–3^ Static structural data from high-resolution structure determination techniques such as X-ray crystallography and cryogenic electron microscopy can provide a detailed picture of these systems. However, the structural data lacks important information regarding biomolecular dynamics^4^ which can occur on a wide range of time scales (the nanosecond time scale for side chain fluctuations and the microsecond time scale for conformational transitions).^5^ In contrast, alternative experimental techniques are able to quantitatively characterize transitions between states,^6^ however, they have a reduced spatial resolution compared to the aforementioned methods. One such technique is single-molecule Förster resonance energy transfer (FRET) spectroscopy or smFRET,^6^ which enables the observation of molecular conformational changes over a broad range of time scales.^7^

FRET is the nonradiative transfer of energy initially absorbed by a “donor” dye to a nearby “acceptor” dye.^8,9^ The energy transferred between a donor and acceptor dye is dependent on the distance between the dyes.^10^ Therefore, the amount of energy transferred between the dyes can be used to provide distance-based information on the molecular conformation at the time of photon emission. Consequently, FRET is often considered to be a “spectroscopic ruler”.^11–15^ FRET measurements can be performed using both ensemble and single-molecule approaches. Ensemble FRET experiments, featuring simultaneous excitation of multiple donors, measures energy transfer for a population of molecules, however this technique suffers from ensemble averaging which can obscure the conformational dynamics underlying the process. Nevertheless, through clever experimental design, valuable conformational information can still be gleaned from this method.^16–18^ On the other hand, the advent of single molecule spectroscopic techniques transformed biophysics into a source of dynamic data on molecular structure as well as function.^19^ SmFRET experiments circumvent ensemble averaging by selectivity exciting donor fluorophores and detecting both donor and acceptor signals at a single molecule level.^20,21^ As a result, distinct conformational states can be detected without the effect of averaging across a large population. The ability of smFRET to resolve conformational transitions depends on the detection method used. Time-correlated single photon counting (TCSPC) can capture fast dynamics on time scales in the nanosecond range, while camera-based smFRET can capture slow molecular rearrangements on the microseconds to seconds time scale.^7^ Regardless of the detection method used, smFRET techniques have become a popular source of spatiotemporal information on the conformational landscape of a molecule and have been applied to a variety of systems from DNA^22^ and RNA^23–25^ to protein folding.^26–28^

The two broad varieties of smFRET experiments are distinguished by how the labeled molecule is isolated from other FRET signals when it is excited. First, surface immobilized experiments fix the labeled molecule to a substrate, expose it to laser light to excite the donor dye, and collect the resulting photon data using integrated cameras.^14^ This experimental procedure uses long exposure times to collect data because photon intensities are calculated by integration over multiple pixels where molecules are identified. The setup allows for multiple molecules to be analyzed simultaneously, but it is limited by the frame rate of the camera which is typically 1 ms^29^ or longer. Despite experimental difficulties arising from surface impacts on dynamics and signal issues from photobleaching, low signal-to-noise ratio (SNR), and photon noise, surface immobilized experiments have been a fruitful area of study.

Second, freely diffusing smFRET methods record photon emission timestamps from labeled molecules as they diffuse through a solution with a confocal laser spot focused inside the solution. Periodically, the path of a molecule will cross the focal region of the laser, where the probability of photon absorption and emission are high. The diffusion rates and concentrations of the molecules in solution as well as the size of the focal region are selected so that the observation of simultaneous excitations of more than one molecule is vanishingly rare within a particular observation time window. The molecules emit bursts of photons as they diffuse through focal beam of the confocal laser spot. Optical filters chosen to correspond to the donor or acceptor photon wavelengths isolate the signal into the two detector channels where photon detectors record arrival times with much greater time resolution than the cameras used in the immobilized experimental setups. Simultaneously, background sources of photons are also reported by the photon detectors.

Freely diffusing smFRET experiments can capture dynamics occurring on faster scales than surface immobilized experiments,^6^ time scales in the nanosecond range opposed to in the microsecond to second range,^7^ because the photon detectors (ex: TCSPC, avalanche photodiodes (APDs), etc.) record with a finer time resolution compared with the cameras used in surface immobilized experiments. Additionally, the potential impacts of the surface on the conformational dynamics observed are avoided when using freely diffusing smFRET,^30–32^ however the short bursts of data provide a challenge for analysis. To combat this challenge, sliding window methods^29^ have been developed to accurately identify the bursts of photons in freely diffusing timestamp data. On the other hand, a Bayesian method for estimating the mean fluorescence from time-correlated, single-photon-counting data has been developed.^33^ Recent research has effectively identified and quantified the within-burst dynamics in single-labeled single-molecule fluorescence lifetime experiments, shedding light on the complex processes occurring at the level of a single molecule.^34^ Safar et al. introduce a Bayesian nonparametric framework for analyzing single-photon smFRET data under pulsed illumination. The method effectively captures system state transitions and photophysical rates while handling uncertainty from various noise sources.^35^ Many other studies also demonstrated accurate estimations of experimental and simulated data, offering a promising approach for unraveling complex molecular interactions in single-molecule studies.^36–38^ Currently, we also have conventional time-correlated single photon counting, which can detect the time-stamping photon arrival times with ps accuracy and is based on the measurement of the detection timings of individual emitted photons with regard to the time of their excitation.^39^

While sophisticated statistical methodology is essential to the analysis of any smFRET experiment, the literature on this topic has primarily focused on surface-immobilized smFRET.^40^ These techniques include histograms,^4^ Gaussian mixture models,^41^ hidden Markov models (HMM),^22,42–44^ and Bayesian nonparametric approaches.^45,46^ Although surface-immobilized smFRET remains a powerful tool for molecular studies, the freely diffusing smFRET technique is gaining in popularity due to its simpler experimental methodology with no need for surface immobilization^47^ and the high time resolution afforded by photon detectors. To further advance the developing fields of smFRET analysis, the ability to realistically simulate the underlying molecular processes in a systematic, controlled, and reproducible manner is a necessity. Additionally, to evaluate new analysis techniques, it is essential to use synthetic data, which enables controlled testing and validation against known ground truth. Several existing smFRET software tools have been developed to generate realistic simulated data.^48^ Some of these tools generate binned photon data with specific distribution characteristics.^49^ Other software, like Fretica^50^ or simFCS 4,^51^ simulates the diffusion of molecules through solution as well as the emission of photons from fluorescent dyes.

In reality, the distances between the dyes on a labeled molecule (dye–dye distance) are constantly changing due to both dye dynamics and the conformational dynamics of the labeled molecule. In a fixed efficiency model a single efficiency value is assigned during each burst implying that conformational motion of the labeled biomolecule occurs fast enough relative to burst length that the resulting FRET signal represents a single time-averaged efficiency value. In these models the heterogeneity of dye–dye distances arising from molecular motion in a freely diffusing labeled molecule is not ignored, but is rather incorporated through the averaged efficiency value. This simplification may be justifiable for highly structured molecules or for conditions that suppress dynamics like low temperatures. However, reductions in molecular structures and greater fluctuations, like those observed in disordered proteins,^52,53^ will invalidate the assumption. Under these conditions, multiple conformations are sampled during a single burst, leading to averaging effects.^6,54^ This is especially true for disordered proteins with reduced secondary and tertiary structure to stabilize the conformations. The flexibility of the molecule leads to a heterogeneous conformational ensemble that poses further challenges to the analysis of experimental data. Ideas from polymer dynamics have been applied to describe the smFRET data from disordered proteins.^55,56^ Biologically important systems often contain large heterogeneity of conformational states,^28,57,58^ so analysis of disordered protein systems has great applicability for future work. The conformational dynamics of proteins is taken into account explicitly in methods such as molecular dynamics simulations,^59–62^ however, in most smFRET data simulation codes, the conformational states are simply represented by single FRET values and single distance values.

To more accurately model the conformational dynamics of a flexible molecule during an smFRET simulation, we integrated overdamped Langevin dynamics, routinely used to represent a simple model for biomolecular dynamics in toy models,^63^ into freely diffusing smFRET timestamp simulation data. While the base PyBroMo software uses Brownian motion to simulate translational diffusion of labeled molecules through the confocal volume without rotational degrees of freedom, our implementation uses overdamped Langevin dynamics to model fluctuations in dye–dye distances arising from the labeled molecule’s conformational dynamics. In the base PyBroMo framework, the effect of dye motion is approximated by assuming rapid, unconstrained diffusion of the dye within its accessible volume, and sampling dye positions randomly within this volume during each simulation step. This simplification is based on the assumption that the dye explores its accessible volume much faster than the time scale of conformational dynamics or photon detection, thereby contributing to a static distribution rather than time-resolved motion. Our integrated approach enables the simulation of the conformational dynamics of the labeled biomolecule on the millisecond time scale or longer which is relevant for many biomolecular systems including intrinsically disordered proteins. The Langevin dynamics simulation produces a trajectory of dye–dye distances for each molecule that conform to an underlying ground truth related to the free energy used in the Langevin dynamics. This addition provides for a more realistic smFRET simulation, particularly important for unstructured proteins or those associated with intrinsic disorder. Although dye dynamics and dye interactions with the host molecule also contribute to distance fluctuations,^64–66^ this implementation focuses exclusively on modeling conformational fluctuations arising from protein flexibility and translational diffusion. The added realism of the integrated overdamped Langevin dynamics will be necessary in the development of new analysis techniques that account for conformational heterogeneity in the labeled biomolecule.

While Langevin dynamics has been used in the past in the context of smFRET, our contribution is different from prior work by integrating (i) physically defined conformational dynamics evolving on an arbitrary free-energy landscape, including those derived from molecular dynamics simulations, with (ii) full 3D Brownian diffusion through a confocal volume and (iii) photon timestamp generation at the detector level. The present work provides the first unified method that couples intramolecular distance dynamics to translational diffusion and PSF-weighted photon emission, enabling realistic, ground-truth synthetic data sets for benchmarking smFRET analysis methods. The originality of our approach therefore lies not in the use of Langevin dynamics itself, but in establishing a complete multiscale simulation pipeline that does not currently exist in the smFRET literature.

The framework presented here provides a general mechanism for incorporating realistic conformational dynamics into freely diffusing smFRET timestamp simulations. The Langevin dynamics implementation presented here serves as a simple and transparent example of this strategy, allowing conformational heterogeneity to be controlled through prescribed free-energy landscapes. Importantly, the framework is not limited to this choice: multidimensional Langevin dynamics and distance trajectories obtained from atomistic MD simulations can be incorporated without altering the overall simulation and analysis pipeline. The contribution of this work is therefore methodological in nature, establishing a flexible foundation for generating realistic synthetic smFRET data sets with known ground truth.

Here, we demonstrate the utility of our approach through a full application to simulated freely diffusing smFRET data. We describe the simulation methods used to generate timestamped photon data, including overdamped Langevin dynamics used to generate the distribution of dye–dye distances which model the internal conformational dynamics of the molecule. Then, two example simulations are described to generate simulated data; one for molecules in a single state and one for molecules that interconvert between two states. A standard analysis for smFRET data is applied using thresholds and Gaussian mixture models were applied to the timestamp data for the single state data using the base PyBroMo software (non-Langevin) and the modified PyBroMo software using the added Langevin dye–dye distances allowing for consideration of the conformational dynamics of the labeled biomolecule of interest. Then, an analysis of the two-state interconversion methodology using the non-Langevin and Langevin timestamp data uses a skewed Gaussian mixture model, as well as change-point analysis and hidden Markov models (HMMs) to assess the dynamics states. We end with a discussion that includes ways to generalize the approach introduced here including an example that incorporates MD simulation data within the developed scheme. The results presented throughout the manuscript illustrate how the method captures conformational heterogeneity in dynamic molecular systems.

In the sections that follow, we outline a unified simulation framework that incorporates both fixed-efficiency and dynamically evolving conformational models within PyBroMo’s freely diffusing smFRET architecture.

## Methods

Before presenting the technical details, we summarize the overall structure of our simulation workflow. As illustrated in Figure 1, the framework consists of four conceptual components: (i) conformational dynamics, (ii) Brownian translational diffusion, (iii) photon timestamp generation, and (iv) downstream smFRET data analysis.

**Figure 1:**
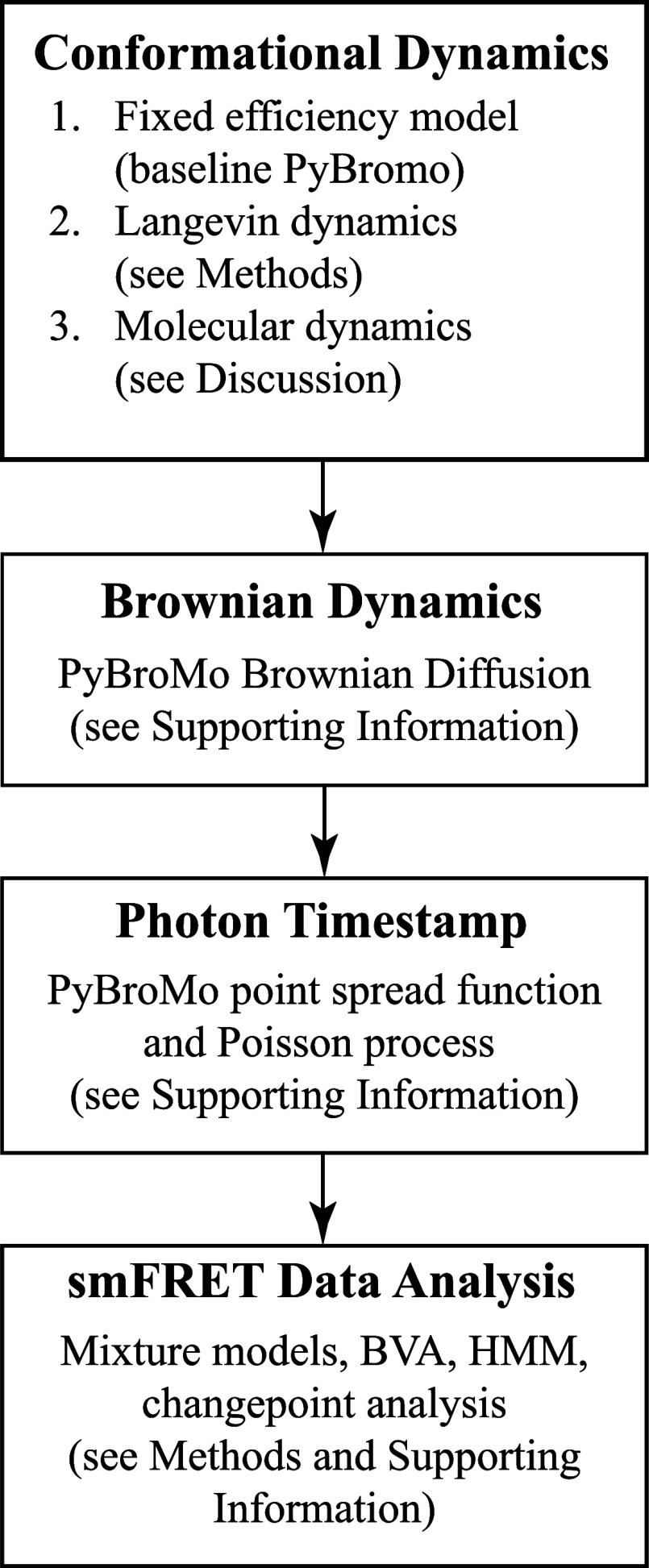
Overview of the smFRET simulation workflow. Conformational dynamics may be modeled using fixed efficiencies, overdamped Langevin dynamics, or MD-derived inputs. These trajectories are combined with PyBroMo-based Brownian diffusion and PSF-weighted Poisson photon emission to generate timestamp data, which are analyzed using mixture models, BVA, HMM, and changepoint methods.

The workflow admits several options for modeling conformational motion. In the fixed-efficiency model (baseline PyBroMo), each molecular population is assigned a constant FRET efficiency and no intramolecular dynamics are simulated. In the Langevin-based model, the dye–dye distance evolves according to overdamped Langevin dynamics on a chosen free-energy landscape, producing time-dependent efficiencies. Finally, we show in the Discussion how molecular dynamics trajectories may be incorporated as an alternative source of distance statistics.

Regardless of the conformational model, each molecule undergoes three-dimensional Brownian diffusion through a confocal point-spread function (PSF), implemented through PyBroMo’s translational diffusion engine (see Supporting Information). Photon timestamps are then generated using a Poisson emission model that accounts for donor and acceptor channels as well as background photons. The resulting timestamp data are subsequently processed using a suite of smFRET analysis tools, including mixture-model inference, burst-variance analysis (BVA), hidden Markov modeling (HMM), and changepoint detection (see Methods and Supporting Information). This overview, together with Figure 1, is intended to provide a conceptual map of the simulation framework. We now describe each component of the workflow.

Timestamps are generated using the freely diffusing smFRET simulation package PyBroMo.^67^ Details on how PyBroMo simulates diffusion of molecules and emission of photons are contained in the Supporting Information (see Simulation Software). To illustrate the impact of conformational dynamics on analysis results, we simulate timestamps with fixed efficiency states and with conformational heterogeneity using Langevin dynamics. We next introduce a Langevin-based conformational dynamics model that generates time-dependent dye–dye distances.

### Overdamped Langevin Dynamics

The use of a static efficiency in the freely diffusing smFRET simulation implies either an underlying static relationship between the two fluorescent dyes labeling the molecule or dynamics between the dyes that are undetectable within a photon burst. Fluctuations in molecular structure, particularly in unstructured proteins, could impact how smFRET data is interpreted. To include conformational heterogeneity beyond the static efficiency assumptions, an overdamped Langevin dynamics simulation is added to generate realistic dye–dye distance fluctuations over the simulation time as a one-dimensional diffusion process within a free energy field.

The Langevin trajectories are calculated according to the Euler-Maruyama method,^68^ where at each time step (δt), the dye–dye distance is updated by calculating the contributions from the distance derivative of the free energy function, *V*(*r*) and a stochastic random contribution. This step update is defined as

1 $$r\left(t+\delta t\right)=r\left(t\right)-\beta D_{L}\frac{dV\left(r\right)}{dr}\delta t+\xi_{L}$$

where *D*_*L*_ is the diffusion coefficient, $\beta =\frac{1}{k_{B}T}$ with *k*_*B*_ being the Boltzmann constant, *T* is the system temperature, and ξ_*L*_ is drawn from a Gaussian distribution of mean 0 and variance 2 *D*_*L*_δ*t* (ξ_*L*_ ∼ *N*(0, 2*D*_*L*_δ*t*)). The diffusion coefficient for the dye–dye distance, *D*_*L*_, is distinct from the Brownian motion diffusion coefficient. The *D*_*L*_ here is the diffusion coefficient associated with the conformational dynamics of protein, which not only depends on the specific protein but also, for the same protein, depends on the location of the attachment of the dyes. In some cases, the interdye distance may fluctuate very fast not visible to the smFRET technique (i.e., fluctuations are much faster than 1 ms). In other cases, the interdye distance may change on a second or minute time scale. The molecules are perturbed by thermal white noise while inside a user defined free energy field.

A FRET efficiency model converts the dye–dye distance trajectories to efficiency trajectories. Two different efficiency models are used for the two example scenarios described in greater detail in the Methods section. However, a constant that is common in efficiency models is the Förster radius, *R*_0_, defined as the distance from the donor dye at which FRET efficiency is 0.5. This *R*_0_ value is specific to the fluorescent dyes used in a smFRET experiment and based on the quantum yield of the donor dye and the spectral overlap of the two dyes. The photon emission simulation takes the efficiency trajectories and uses the efficiency value at the time of photon emission and uses it to determine the ratio of donor and accetor photons. The background timestamp generation is independent from the Langevin dynamics and contributes to the Poisson distributed background timesteps as before. Next, we describe the two example simulations to compare simulated data with and without conformational heterogeneity from Langevin dynamics.

### Molecules in a Single State

To demonstrate the generation of timestamps using the Langevin dynamics conformational trajectories, a simple example system of molecules in a harmonic free energy field is simulated for three independent simulations with all parameters held constant. A Gaussian distribution of distance is a common assumption in polymer physics, though other distributions have been explored to account for more complex system interactions.^69–71^ The harmonic function, *V*_*H*_ is defined as

2 $$V_{H}\left(r\right)=\frac{k_{H}}{2}{\left(r-r_{c}\right)}^{2}$$

where *k*_*H*_ is the harmonic force constant, and *r*_*c*_ is the center of the harmonic well. 100 molecules are contained in a simulation box with lengths *L*_*x*_ = *L*_*y*_ = 8 μm, *L*_*z*_ = 12 μm. The Brownian diffusion coefficient, *D*_*B*_, is set to 30 μm^2^/s for all molecules. Diffusion coefficients for proteins in water are on the order of 20–200 μm^2^/s^72,73^ making this value on the slower side of the spectrum. A faster diffusion rate would decrease the average duration of a burst of photons as the molecule traversed the focal spot in a shorter period of time. The Gaussian point spread function (PSF) is centered in the simulation box with a σ_*x*_ = σ_*y*_ = 0.3 μm, and σ_*z*_ = 0.5 μm. Three independent simulations are run for 10s each with a time step, δ*t*, of 50 ns. For timestamp generation, a maximum emission rate of 200,000 counts per second (cps) is used in all the simulations, as well as a background rate of 1200 cps for the acceptor channel and 1800 cps for the donor channel. The cps values will be kept consistent for all simulations used in this work.

For the Langevin dynamics parameters, the thermodynamic coefficient β is 1.339 (kcal/mol)^−1^ which corresponds to a relatively high temperature of 378 K for large thermal fluctuations. The Langevin diffusion coefficient, *D*_*L*_, is 1300 Å^2^/ns. This is a rapid diffusion employed to explore the free energy well in a short trajectory. The harmonic function is defined by eq 2 with the coefficient *k*_*H*_ set at 0.025 (kcal/(mol Å^2^)) with the center of the harmonic well at 40 Å for 50 of the molecules, and at 65 Å for the 50 molecules. eq 4 is used to convert the distances to efficiencies. In efficiency conversions, an *R*_0_ of 56 Å is used.

A short trajectory of Langevin dye–dye distances is shown in Figure 2. The molecules in each population oscillate in the harmonic free energy field over time, with a probability of some dye–dye distance, *P*(*r*), following the relation

3 $$P\left(r\right)=\frac{1}{2\sqrt{\frac{2\pi }{\beta k}}}\left(e^{-\beta V_{H1}\left(r\right)}+e^{-\beta V_{H2}\left(r\right)}\right)$$

where *V*_*H*1_ and *V*_*H*2_ are the harmonic free energy functions applied to the two molecule populations in the Langevin dynamics simulation.

**Figure 2:**
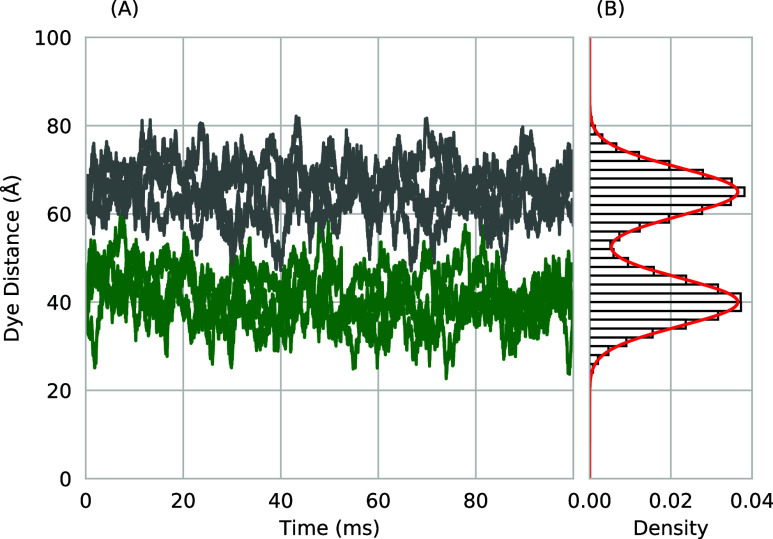
(A) A portion of a trajectory of Langevin dynamics for the dye–dye distance of 4 molecules in a harmonic free energy centered at 40 Å (colored green) and 4 molecules in a harmonic free energy centered at 65 Å (colored gray). (B) A histogram of the dye–dye distances for the combined populations along with the analytic solution for the distribution in red.

For the harmonic free energy simulations, an efficiency model developed for conformationally heterogeneous proteins^53^ relating the dye–dye distances to FRET efficiency is

4 $$E=\frac{1}{1+0.975{\left(\frac{r}{R_{0}}\right)}^{2.65}}$$

where *r* is the dye–dye distance and *R*_0_ was 56 Å. This efficiency model which uses a power of inverse 2.65 instead of the classical Förster inverse sixth-power dependence is used in part because this work focuses on the heterogeneous conformations of proteins and we wanted to better reflect the behavior observed in conformationally heterogeneous systems. In these systems, the donor and acceptor dyes experience restricted motion meaning the assumptions of ideal dipole–dipole interactions and isotropic averaging do not hold. Kuzmenkina et al.^74^ demonstrated this deviation previously using smFRET data on disordered biomolecules. Since our simulations are specifically designed to capture the dynamic conformational heterogeneity of proteins, the 2.65 exponent provides a more realistic mapping between dye separation and FRET efficiency under these conditions. In using this exponent, we also showcase that our approach can accommodate alternative efficiency models (in the next example, the more standard inverse 6th power efficiency model is used). The FRET efficiencies used for photon generation are 0.41 and 0.71, which corresponded to eq 4 applied to distances of 40 and 65 Å, respectively. The distances matched the centers of the harmonic functions used in the Langevin dynamics simulations. The other photon generation parameters for maximum emission rate and background noise were held constant.

To compare the results of the simulations with the new Langevin dye–dye distance trajectories with the fixed efficiency assumption, three sets of simulated timestamps were generated with the base (non-Langevin) PyBroMo. These simulations used the same number of molecules, and other Brownian motion parameters for diffusion coefficient, simulation box, PSF, and background photons as described above. 50 molecules had an efficiency of *E* = 0.71 while the other 50 had an efficiency of *E* = 0.41. These efficiency values correspond to eq 4 applied to the harmonic centers from the Langevin dynamics, 40 and 65 Å respectively.

### Molecules with Interconversion Between Two States

The harmonic free energy Langevin simulations described above approximate a system where the dye–dye distance fluctuates around a single state for the duration of the simulation. However, biophysical intuition as well as experimental smFRET data suggest that many biomolecular systems correspond to two or more interconverting conformational states at equilibrium.^75^

To simulate a system that dynamically moves between different states, a bistable free energy with two symmetric wells are applied to a system of molecules in the Langevin dynamics simulation. 90 molecules were simulated using the Langevin dynamics simulation with the same *D*_*B*_, time step length, simulation box, and PSF as previously defined in single state molecule simulations. This bistable function, *V*_*B*_(*r*), is defined as

5 $$V_{B}\left(r\right)=\frac{k_{B}}{4}{\left({\left(r-r_{C}\right)}^{2}-W^{2}\right)}^{2}$$

where *k*_*B*_ is the bistable force constant set at 10^–4^ (kcal/(mol Å^2^)). The location of the center of the bistable function, *r*_*C*_, is set in this example at 50 Å, and *W* is the offset from the center where the free energy wells were located, set at 15 Å. The locations of the bistable minima is at *r*_*c*_ ± *W*, or 35 and 65 Å with a barrier height of 1.265625 kcal/mol. Using the bistable free energy function, a Langevin molecule will explore a local free energy energy well until a large enough energetic contribution from the white noise in the Langevin dynamics gives the molecule the energy to overcome the energy barrier and explore the other well. A Langevin diffusion coefficient of *D*_*L*_ = 0.002 Å^2^/ns is used. This value is selected for slower dynamics.

FRET efficiency is modeled using the commonly used relation:

6 $$E=\frac{1}{1+{\left(\frac{r}{R_{0}}\right)}^{6}}$$

where *r* is the dye–dye distance and *R*_0_ = 56 Å, as before. The efficiency model in eq 6 is based on a point dipole–dipole approximation and is widely used in smFRET data analysis.^14^ The dye rotational dynamics is assumed to be considerably faster than protein conformational dynamics. In order to gather a sufficient amount of data for analysis, a total of approximately 20 min of simulated smFRET data is generated.

A short dye–dye distance trajectory using the bistable function is shown in Figure 3. We see the dye–dye distances fluctuate within one of the free energy wells for some period of time before occasionally overcoming the energy barrier and crossing into the other well, thus switching states. The distribution of dye–dye distances for the bistable Langevin simulation follows the relation

7 $$P\left(r\right)=\frac{1}{Z}e^{-\beta V_{B}\left(r\right)}$$

where the partition function for the bistable free energy, *Z* = ∫_0_^∞^ *e* ^–*βV*_*B*_(*r*)^d*r*, normalizes the probability density to 1. A lower temperature, *T* = 300 K, is used as compared to Example 1, with β = 1.679 (kcal/mol)^−1^. The lower temperatures decrease the magnitude of thermal fluctuations for each time step so the molecule will explore the local well long enough to emit sufficient photons for the state to be identifiable.

**Figure 3:**
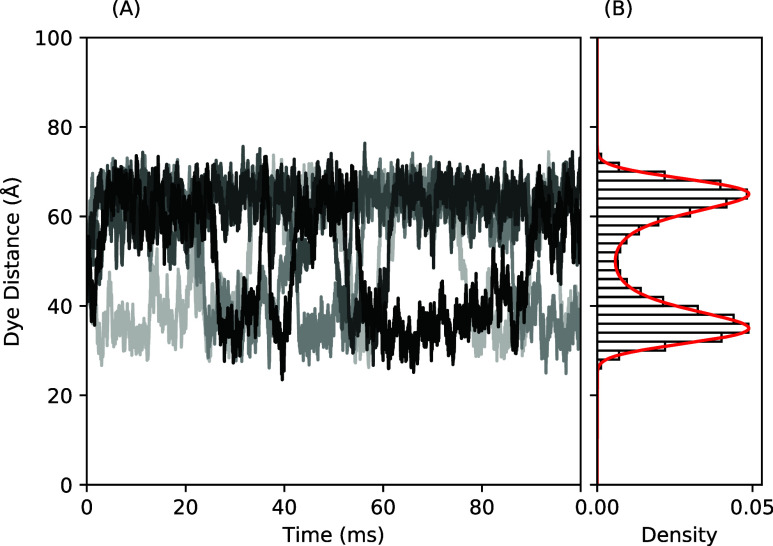
(A) A portion of Langevin dynamics trajectories for the dye–dye distance of 5 molecules moving in a bistable free energy field centered at 50 Å with minima at 65 Å and 35 Å. (B) A histogram of the dye–dye distances is shown with the theoretical probability shown as a red line.

The analytical transition matrix of the bistable Langevin simulation, *T* ^(0)^, between different states is related to the transition rate matrix, *Q*, by

8 $$T^{\left(0\right)}=\exp\left(\tau Q\right)$$

9 $$Q≔\left[\begin{array}{ll} Q_{1,1} & Q_{1,2}\\ Q_{2,1} & Q_{2,2} \end{array}\right]$$

where τ is the lag time between state determination measurements. The entry *Q*_*i*, *j*_ represents the transition rate from state *i* to state *j*. The transition rate between two nonidentical states (here reactant, *R* and product, *P*) is calculated using relations from Berezhkovskii and Szabo,^76^

10 $$Q_{R\to P}=\frac{1}{(\int_{-\infty }^{x*}e^{-\beta V\left(x\right)}dx)\left(\int_{R}^{P}e^{\beta V\left(x\right)}dx/D\left(x\right)\right)}$$

where the integration limit x* is the peak of the barrier at 50 Å, *V*(*x*) is the free energy, *D*(*x*) is the position dependent diffusion coefficient, and $\beta =\frac{1}{k_{B}T}$ . Substituting in the bistable function, *V*(*x*) = *V*_*B*_(*x*) and the constant Langevin diffusion coefficient, *D*(*x*) = *D*_*L*_, the transition matrix can be computed theoretically as,

11 $$T^{\left(0\right)}=\left(\begin{array}{ll} 0.968 & 0.032\\ 0.032 & 0.968 \end{array}\right)$$

Here, *Q*_1, 2_ = *Q*_1 → 2_ and *Q*_2,1_ = *Q*_2 → 1_ (which are both equal given the symmetry of *V*_*B*_(*x*) with respect to the two states) are calculated from eq 10. Also *Q*_1,1_ = −*Q*_1,2_ and *Q*_2,2_ = −*Q*_2,2_. Finally, *T*(0) is calculated from *Q* using eq 8.

In addition to the 90 molecules in the bistable Langevin simulation, 10 molecules were kept in a constant “donor only” state of *E* = 0. Donor only states are present in experimental data and represent molecules where only the donor dye is attached, with no FRET possible. The donor only population represents a phenomenon of imperfect labeling where the acceptor dye is not present as this is a source of error encountered by experimenters. This is a static state and does not adequately describe other similar error sources like photoblinking, causing a temporary donor only signal.

To provide a comparison with the bistable Langevin timestamps, non-Langevin timestamps were generated that simulated dynamic state switching. This is done by generating two timestamp traces of approximately 20 min in length, using the same parameters for Brownian motion as the bistable Langevin data. One set of timestamps used a fixed high efficiency state of *E* = 0.944, while the other used a fixed low efficiency state of *E* = 0.290. The efficiencies correspond to eq 6 using the locations of the well minima, *r*_*C*_ = 35 Å for the high efficiency state and *r*_*C*_ = 65 Å for the low efficiency state Also, a Förster radius of *R*_0_ = 56 Å was applied in all the efficiency calculations. Again, the Brownian motion simulations parameters of Brownian diffusion constant, simulation box size, PSF, and background photons were the same for the non-Langevin simulation as with the Langevin dynamics simulations above.

Transitions between states were simulated by drawing residence times from an exponential distribution with an average residence time of 31.126 ms. The trajectory of an efficiency state evolves like a step function alternating between the two states. This residence time leads to a transition matrix for the non-Langevin data that closely matches the transition rate matrix generated from the bistable free energy. Using these residence times, a set of timestamps is created that switched between the two efficiency states, also 20 min in overall length.

A comparison of the results from three analysis methods performed on the dynamic state model simulation using non-Langevin and Langevin timestamps are contained below.

## Results

Techniques for simulating freely diffusing smFRET experiments are valuable, in large part, because they allow researchers to evaluate statistical methods using realistic data with a known ground truth.With this in mind, we present a standard analysis of the timestamp data produced from the parameters described in the Methods section.

### Comparison of Single State Simulations

The timestamp data generated by both non-Langevin and Langevin simulations was in the form of a column of ordered timestamps when a photon was detected. Additional columns label the channel that detected the photon (donor or acceptor), and add a label to identify the molecule that emitted the photon. This molecule identifier would not be available in experimental data, but is information that is available in the simulation.

Data analyses of freely diffusing smFRET experiments typically begin by binning and thresholding the raw photon time stamp data.^40,77^ The time bin size needs to be long enough to collect sufficient data such that the signal from the fluorescent dyes can be distinguished from the noise contributions. Conversely, the bin size needs to be small enough so that the FRET signal is only from one molecule. The specific choice of time bin length will be dependent on background noise rates, molecule diffusion rates, and confocal beam size, on the order of 1 ms.^29^ In our analyses, we use a typical experimental bin width of one millisecond. For a given experiment, let *I*_*t*_^D^ and *I*_*t*_^A^ denote the photon counts in the donor and acceptor channels during time bin *t* and define the combined count *I*_*t*_^C^ = *I*_*t*_^D^ + *I*_*t*_^A^. We restrict our analyses to those time bins with combined count exceeding 40 photons. Based on the simulation parameters that are used, a combined photon count at or above this magnitude indicates that the signal is very likely from a molecule diffusing across the focal beam and thus the proportion of photons in the acceptor channel reflects the molecule’s conformational state. Threshold also ensures that our estimates of the efficiencies within each time bin are not excessively variable due to low counts. No single method to determine photon thresholds has been universally accepted.^78^ In the literature, there are a number of heuristics for choosing the threshold and many alternative approaches to identifying the diffusion of a molecule across the focal beam.^79–81^

Central to our analysis are the estimates of efficiencies within each bin, which we refer to as *apparent efficiencies*.^14^ The apparent efficiency within bin *t* is defined as the proportion of the total photon count from that bin which was detected in the acceptor channel:

12 $${Ê}_{t}=\frac{I_{t}^{A}}{I_{t}^{A}+I_{t}^{D}}$$

When analyzing real smFRET experiments, estimation of efficiencies should also take into account the so-called γ factor, which accounts for the difference in quantum yields of the donor and acceptor dyes as well as the difference in photon detection efficiencies of the donor and acceptor channels.^82,83^ Other experimental error sources, include adjustments for direct excitation of the acceptor dye from laser and leakage of acceptor photons into the donor channel.^84^ These adjustment is not necessary for our analysis because the smFRET simulations in this article were run with equivalent quantum yields and equivalent detection efficiencies.

We analyze the simulated smFRET experiments using a simple histogram of the apparent efficiencies as well as a Gaussian mixture model fit to the apparent efficiencies. The histogram approximates the marginal distribution of efficiencies. It provides an idea of the relative amount of time a molecule spends at each efficiency and whether there exist easily distinguished conformational states. In comparison to a histogram-based analysis, the analysis based on a Gaussian mixture model provides more quantitative information related to hypothesized latent conformational states. We suppose that there is a latent conformational state *s*_*t*_ ∈{1, ..., *K*} associated with each time bin *t* and that these latent conformational states are independent and identically distributed with probabilities π_1_, ..., π_*K*_. Given that *s*_*t*_ = *k*, we suppose that the apparent efficiency *Ê*_*t*_ follows a Gaussian distribution with mean μ_*k*_ and variance σ_*k*_^2^. The smFRET simulations were run with *K* = 2 conformational states, and we take this as given. We compute the maximum likelihood estimates of the unknown parameters via an expectation-maximization algorithm^85^ as implemented in the mixtools package^86^ in R.^87^

Figure 4 compares the non-Langevin and Langevin simulations in terms of apparent efficiencies and the corresponding dye–dye distances. Figure 4A, based on the non-Langevin simulation, shows the estimated two-component Gaussian mixture density (in solid black) on top of a histogram of the apparent efficiencies. The dashed lines represent the (weighted) densities of the estimated component distributions. The low efficiency component has a mean of 0.42, a standard deviation of 0.07, and a mixture weight of 0.62. The high efficiency component has a mean of 0.70, a standard deviation of 0.05, and a mixture weight of 0.38. The vertical red arrows are placed at the true efficiency values used in the simulation. Figure 4B shows the corresponding histogram, densities, and arrows after a transformation to the distance space. The probability distribution of distances is converted to a probability distribution of efficiencies through a change of variable based on the efficiency model in eq 4 The conversion is over the apparent efficiencies which represent an average over 1 ms of data. This averaging has the potential to obscure faster internal dynamics that occur within 1 ms time bin.

**Figure 4:**
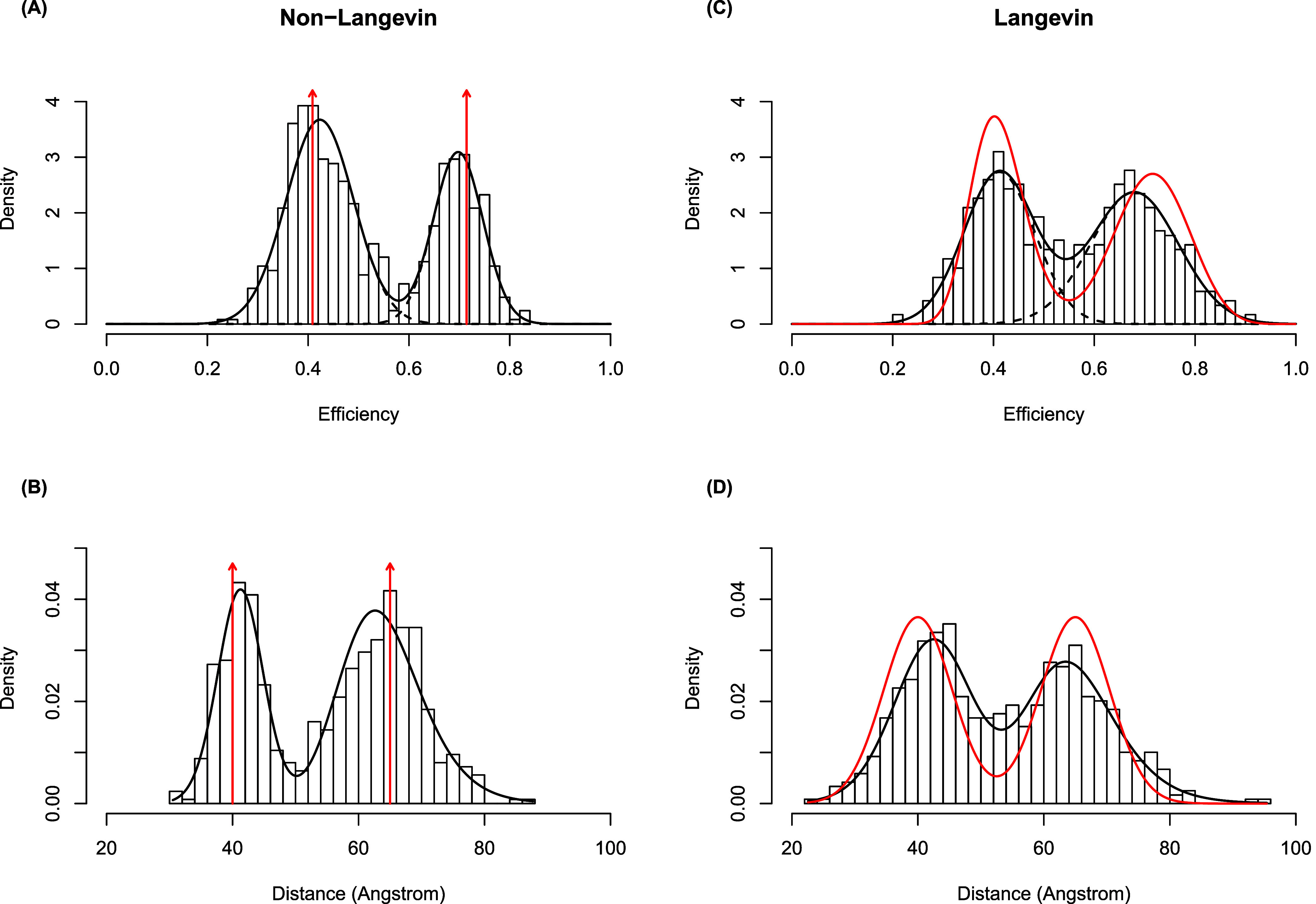
(A) The estimated Gaussian mixture density (solid black line) from the non-Langevin simulation on top of a histogram of the apparent efficiencies along with the two true efficiencies (red vertical arrows). (B) The corresponding plot in the distance space. (C) The analogous efficiency plot for the Langevin simulation. (D) The analogous distance plot for the Langevin simulation. The density curves in red shown in C & D are the “ground-truth” analytical distributions of efficiency and distance, respectively, associated with the Langevin model. The vertical arrows in A & B are the corresponding distributions in the non-Langevin model that represent Dirac delta functions.

In contrast to Figure 4A,B, which are generated from the Non-Langevin simulation, Figure 4C,D are based on the Langevin simulation. For a better comprehension of the graphs, Langevin simulation results Figure 4C–D contain density curves showing the true, nondegenerate theoretical distribution of efficiencies (or distances) in place of vertical red arrows at two actual efficiencies (or distances) in Figure 4A–B. The two-component Gaussian mixture as specified by eq 3 is the theoretical distribution in the distance space. Again, using the same equation, eq 3, and changing the variables from efficiency to distance, we calculated the theoretical distribution in the space of efficiency. The low-efficiency component in Figure 4C has a mean of 0.41, a standard deviation of 0.07, and a mixture weight of 0.48, whereas the high-efficiency component has a mean of 0.68, a standard deviation of 0.09, and a mixture weight of 0.52. Figure 4 displays the underlying Langevin dye–dye distance distribution as a red line Figure 4C–D. Compared to the Langevin simulation timestamp analysis, which exhibited a larger distribution with more overlap, the separate peaks shown in the non-Langevin timestamp analysis revealed less overlap in the distribution of the two populations. Due to the overlap between the underlying distance distributions of the Langevin dynamics for the two populations, this little discrepancy is understandable. Overall, the research demonstrates that the addition of overdamped Langevin dynamics to a straightforward situation results in timestamps that include important data from the underlying distance distribution, such as the locations of efficiency peaks. We note that we do not expect the analytical distributions and the simulation data to be identical since what we describe as the analytical distribution here is simply based on the conformational dynamics of a fixed protein in a theoretically idealized case, where both the Brownian dynamics and the shot noise process are both ignored. As a result, even in the case of the non-Langevin model, we expect the broadening of the observed distributions although the analytical distributions are Delta functions.

### Comparison of State Interconversion Simulations

Next, we describe two more sophisticated analyses that account for additional realistic features included in the simulation, like donor-only particles and dynamic state changes. A histogram based analysis as well as analyses to infer state dynamics were performed. Again, the non-Langevin and Langevin timestamps generated used the simulation parameters that contained information consistent with the simulation parameters that was detectable by the analyses.

#### Skewed-Gaussian Mixture Model

We again analyze the non-Langevin and Langevin timestamps through mixture models. This time, we fit three component skewed-Gaussian mixture models to the timestamps generated from Example 2. Adding a third component is necessary because these simulations include donor-only molecules, leading to a low FRET peak. The skewed-Gaussian distribution has density

13 $$\frac{2}{\omega }\phi \left(\frac{x-\xi }{\omega }\right)\Phi \left[\alpha \left(\frac{x-\xi }{\omega }\right)\right]$$

where ϕ and Φ are the density and distribution functions of a standard Gaussian random variable, ξ is a location parameter, ω is a scale parameter, and α is a shape parameter.^88,89^ This more flexible parametric family allows us to adequately model skewed distributions. Apparent efficiency distributions which lie near the boundary of the unit interval, including the low FRET peak, typically exhibit strong skewness. We compute the maximum likelihood estimates of the unknown parameters via an expectation-maximization algorithm as implemented in the mixsmsn package.^90^

The results appear in Figure 5, which compares the non-Langevin and Langevin simulations in terms of apparent efficiencies and the corresponding dye–dye distances. Figure 5 is analogous to Figure 4, except here they depict the results of the skewed-Gaussian mixture model. The skewed Gaussian mixture analysis was able to recover the location of efficiency peaks from the timestamp data reasonably well for both the non-Langevin and Langevin data, as well as the donor-only peak. Again, the efficiency states for the non-Langevin simulation timestamps showed higher, more well-defined peaks with less overlap than the Langevin simulation timestamps, consistent with the point mass distribution in the distance. This method aggregates all the timestamp information over time into a histogram, losing temporal information about switches between states.

**Figure 5:**
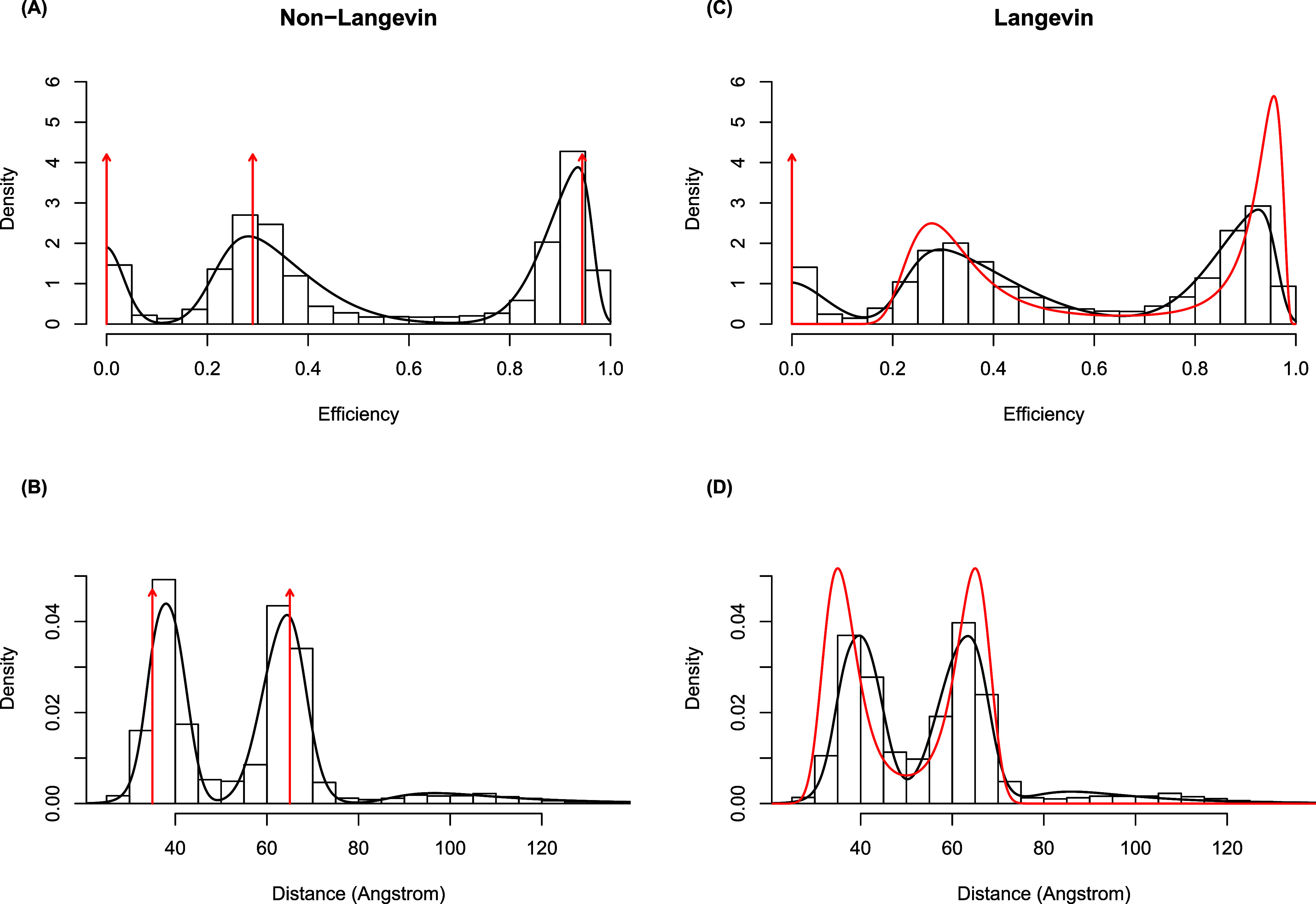
(A) The estimated skewed-Gaussian mixture density (solid black line) from the non-Langevin simulation on top of a histogram of the apparent efficiencies along with the true efficiencies (red vertical arrows). (B) The corresponding plot in the distance space. (C) The analogous efficiency plot for the Langevin simulation. (D) The analogous distance plot for the Langevin simulation. The density curves shown in red in C & D are the true, nondegenerate theoretical distribution of efficiencies (or distances).

Although mixture-model analyses can compare how sharply or broadly efficiency distributions appear in the simulated data, it is important to emphasize that discrete-state inference tools do not recover the underlying continuous physical dynamics. As recently demonstrated by Schweiger, Saurabh, and Pressé,^91^ such models impose artificial segmentation and state structure onto trajectories that originate from continuous diffusive motion. Therefore, differences observed between non-Langevin and Langevin simulations under mixture-model analysis should not be interpreted as evidence that the non-Langevin model “fails” in an inverse-modeling sense. Instead, the key distinction is that non-Langevin simulations produce efficiency distributions that are unrealistically narrow and noise-free, while in contrast Langevin simulations reproduce the stochastic broadening and heterogeneity characteristic of experimental smFRET bursts. Accordingly, the motivation for introducing Langevin dynamics is improved realism of the simulated observables, rather than validation through discrete-state inference. The next two analysis methods will explore the state switching in the timestamp data with more depth.

#### Burst Analysis

In the previous histogram analyses, a threshold placed on the binned timestamp data was used to identify the bins with FRET signal “bursts” that occur when a molecule diffuses through the focal spot and background noise. More sophisticated methods have also been developed to identify bursts of FRET signal in timestamp data.

FRETBursts^78^ is used to identify bursts with the simulated data from example 2. This software applies the “sliding window” search algorithm.^80^ For our initial search, we applied a window of 200 consecutive timestamps with a threshold of 40,000 cps to that window. After the initial search, only bursts with at least 100 photons are selected. The selection of parameters for a burst search can be somewhat arbitrary, though the general approach was to pick a threshold higher than background.

A burst variance analysis (BVA)^92^ method is performed to compare the non-Langevin and Langevin timestamp data to determine if the non-Langevin or the Langevin timestamp data exhibits dynamic FRET fluctuations. The BVA method with the FRETBursts software is implemented based on Torella et al.,^92^ where each burst, *i*, is segmented into a number of consecutive and nonoverlapping windows of *n* photons to calculate the standard deviation of estimated efficiencies within the burst (σ_*i*_).^92^ For a static molecule, the standard deviation of FRET follows a predictable analytical relationship with its mean FRET value. In contrast, molecules undergoing dynamic FRET fluctuations exhibit a larger standard deviation due to conformational changes occurring within the measurement time scale.^92^ Here, each burst was split into sub-bursts of *n* = 10 photons in order to calculate the standard deviations, σ_*i*_ efficiencies in Figure 6. A 2D histogram with a kernel density estimate (KDE) smoothing presents the smooth contour plots of the distribution of efficiencies and σ_*i*_. A dashed line represents the σ of a binomial distribution for the size of the sub-burst. State peaks that occur on the dashed line are static, while state peaks above the dashed line contain interconversion of states within a burst. In both data sets, the two FRET states at 0.29 and 0.94 are visible with the small peak from the donor only contribution visible in the lower left of each plot. Both state peaks in the non-Langevin analysis are centered on the dashed line, indicating negligible dynamic heterogeneity, as expected. Conversely, both state peaks of the Langevin data are near the dashed line, but slightly above, indicative of a small dynamic heterogeneity. More visible differences arise in the areas representing interconverting states that arc between the main peaks and have higher variances. In the non-Langevin data, the interconverting arc is less populated, while the Langevin data shows a higher population for this high-variance region; although it still represents a small population compared to the two main peaks. These observations are consistent with the differences in the underlying process of state interconversion for the Langevin and non-Langevin data. In the non-Langevin model, the states interconvert in discrete states and fewer bursts would capture this interconversion as compared to the Langevin model, where the states interconvert more gradually. The slight shift in the two main peaks of the Langevin model is also explained with the fact that these states are not associated with fixed distance/efficiencies. In other words, one expects dynamic fluctuations within each of the two major states. We have done a similar BVA analysis based on Example 1 (single state) data that shows the same static behavior for both Langevin and non-Langevin models as expected, confirming the accuracy of the BVA approach to distinguish between the static and dynamic behavior (see BVA: Single State and Figure S1 in Supporting Information).

**Figure 6:**
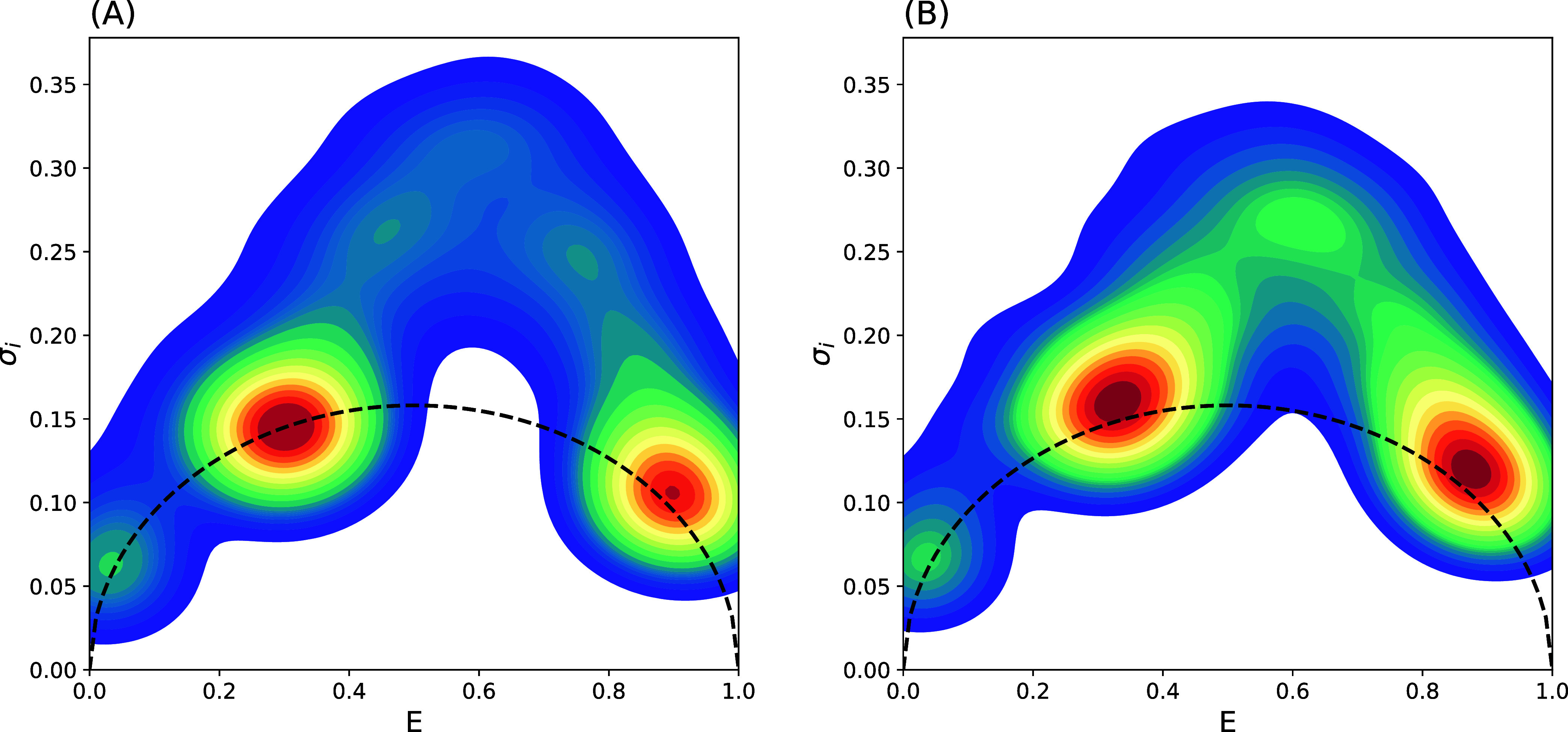
Burst variance analysis (BVA) to compare the (A) non-Langevin and (B) Langevin data. Each burst was split into sub-bursts of 10 photons to calculate the standard deviation. A kernel density estimate (KDE) is used to present smooth contours the scatter plot of efficiency and variance of efficiency for each burst. The dotted line shows the standard deviation of a binomial distribution for 10 samples.

A different metric called FRET-two-channel kernel-based density distribution estimator (2CDE)^93^ estimates the density of photons in each channel for each photon in a given burst. Analysis of FRET-2CDE in the Supporting Information shows a similar difference between the non-Langevin and Langevin data from Example 2 with the arc of interconverting states between the main peaks being thicker with higher density of photons in the Langevin data (see FRET-2CDE and Figures S2 and S3 in Supporting Information).

#### HMM Analysis

We analyze the example 2 timestamp data using a hidden Markov model (HMM).^94^ While HMMs are widely used for detecting state transitions in smFRET data, they assume memoryless transitions and are sensitive to the choice of time binning. Recent developments in continuous-time HMMs aim to better capture fast kinetics and binning-independent behavior.^95^ Here, we specifically consider only the time-bins which are above a threshold (where the total photon count is above 40). In contrast to surface immobilized smFRET, in freely diffusing smFRET experiments the molecule is only sometimes in front of the focal spot.^44,78^ Other researchers have used a burst search algorithm^78^ to identify the parts of the trajectory when the labeled molecule is in the focal spot. A “window search” algorithm^80^ is employed in a burst search analysis. For this analysis, we define a burst region as a set of consecutive time bins such that for each of them, the total photon count is above the threshold. We then evaluate the sequence of apparent efficiencies for each burst region. To perform dynamical analysis and detect transitions between the different FRET states, we treat the sequence of apparent efficiencies from each burst region as an independent time-series to be modeled with the HMM,^94,96^ where the HMM parameters are constant for all the independent time-series. We fit the apparent efficiencies using two hidden states, and assume they are normally distributed conditionally on each state. We have restricted the HMM model to two states with distribution analysis above a threshold of 40 photon count from Example 2. Python’s hmmlearn^97^ package was deployed to fit the HMM.

For the data generated using Langevin dynamics, the average photon burst region duration is 2.18 bins of 1*ms*. We fit the HMM using a total of 30053 such burst regions and obtain a transition matrix estimate

14 $$T^{L}=\left(\begin{array}{ll} 0.960 & 0.040\\ 0.056 & 0.944 \end{array}\right)$$

corresponding to two Gaussian states, for which we estimate means, μ_1_ = 0.321, μ_2_ = 0.883, and variances σ_1_^2^ = 0.029, σ_2_^2^ = 0.004, respectively.

For comparison, we analyze the data generated using non-Langevin dynamics, where the average photon burst duration is 2.20 bins of 1*ms*. We fit the HMM using a total of 31354 such burst regions. Fitting the data results in a transition matrix:

15 $$T^{\mathrm{NL}}=\left(\begin{array}{ll} 0.956 & 0.044\\ 0.061 & 0.939 \end{array}\right)$$

corresponding to two Gaussian states, with means, μ_1_ = 0.291, μ_2_ = 0.910, and variances σ_1_^2^ = 0.025, σ_2_^2^ = 0.002, respectively.

Qualitatively, the measured transition matrices for both the Langevin and non-Langevin models look reasonably similar to the analytical transition matrix in eq 11. The Langevin model, however, results in a transition matrix that is slightly closer to the ground truth. This is verified using a careful quantitative analysis of the difference between the known and measured transition matrices along with error analysis, presented in Supporting Information (see Quantifying Error for HMM Analysis).

A visualization of the transitions using changepoint analysis is presented in Supporting Information, and shows reasonable qualitative agreement between Langevin and non-Langevin simulations (see Changepoint Analysis and Figure S4 in Supporting Information). From these results we can infer that the Langevin dynamics module produces timestamps that include dynamic state changes in a controlled and realistic manner.

## Discussion

The new inclusion of Langevin dynamics allows for generation of more realistic smFRET data consistent with what one expects to observe from freely diffusing smFRET experiments of molecules with flexible conformational states, where a fixed FRET efficiency or dye–dye distance does not provide a reasonable approximation, compared to non-Langevin dynamics. The comparison between the Langevin and non-Langevin models here was not to show the superiority of the Langevin method over the non-Langevin method as the Langevin method is considered an improvement simply because it is more realistic. Instead, the comparison was made to show the newly added Langevin model can be recovered from the data using typical data analysis methods at least as accurately as the original non-Langevin model and that it is compatible with the PyBroMo software.

In the results presented above, the data from two example simulations using the Langevin and the non-Langevin methods were analyzed using typical methods applied to experimental smFRET data. In the first example, a simple model for a flexible molecule where the dye–dye distances evolve dynamically using a Langevin simulation method with a harmonic free energy, generated a distribution of distances and FRET efficiencies in a physically justifiable way. In the second example, the same Langevin simulation method generated dye–dye distances with bistable free energy to model a system that intercoverts between two states. Both examples are compared with simulated data generated with non-Langevin methods for single and bistable states with other parameters that match the Langevin simulations as closely as possible. This is done as a validation exercise to identify any unintended artifacts from the new conformational heterogeneity when compared with the non-Langevin data using standard analysis methods including applying photon count thresholds, binning data over 1 ms, creating histograms, and fitting HMMs to estimate state transitions. Also, this allows for a comparison of some commonly used freely diffusing smFRET data analysis techniques to detect differences in the internal conformational dynamics.

Our results demonstrate both, agreement between the Langevin and non-Langevin results as well as reasonable accuracy in reproducing some of the major parameters of the underlying simulation. For instance, the histogram analyses reproduced the locations of efficiency peaks used as Langevin simulation parameters, in approximately equal proportions for the dye–dye distance distributions. Additionally, the HMM estimated similar transition matrices for the Langevin and non-Langevin timestamp data. Both Langevin and non-Langevin data agree quantitatively with the ground truth within the predicted error limits for their respective analyses.

Qualitative differences are present between the non-Langevin and Langevin timestamp data in the histogram analysis. The histograms of the Langevin timestamp data showed broader distributions of the efficiency states, in general. The comparatively narrow distributions of efficiencies from the non-Langevin timestamp data were due solely to the Brownian motion of the molecule through the PSF, but the underlying efficiency distributions are point masses. Both Langevin and non-Langevin simulation methods contained the same Brownian motion and PSF parameters so any broadening of the efficiency distribution for the Langevin timestamp data can be attributed to the ensemble of dye–dye distances from the Langevin dynamics. Similarly, the BVA also showed differences in both the positioning of the two major peaks and the population of the interconverting states. The non-Langevin results showed peaks centered along the binomial variance line indicating negligible dynamic heterogeneity, as expected. In contrast, the peaks for the Langevin data were slightly above the binomial variance line, suggesting small interconversions between states and that the two major states are slightly dynamic due to local protein conformational fluctuations. Additionally, the interconverting arc between peaks was more populated in the Langevin data, consistent with the expectation that Langevin dynamics introduce gradual transitions rather than discrete state jumps.

It is of note that the conversion between efficiency and distance, as done in the histogram analysis, is potentially impacted by averaging of time bins and generally is nonlinear. We see from the agreement in Figure 4 that the averaging over 1 ms of simulated data gives a reasonable approximation of the underlying dynamics. Qualitative observations, like relative peak heights, can change after conversion. This is most obvious in Figure 5, where the two FRET states have different efficiency peak heights but the peaks of distance histograms (and underlying distribution for the Langevin simulation) are the same height. The two efficiency models used in this paper have qualitative similarities but each model required its own conversion. FRET is most accurate near the *R*_0_ value for the dye pair, with efficiency data becoming more distorted as it approaches zero or one. Accurate conversion of efficiency histogram states into distance is required to infer the underlying state information.

Beyond validation, the qualitative similarity in results from analysis of both non-Langevin and Langevin data implies the need for more sophisticated analysis methods. Despite the stark differences in the ground truth of dye–dye distances, it would be difficult to identify the Langevin results from the non-Langevin results when presented in isolation. Some identifiers of the underlying ground truth are present, like the wider spread of apparent efficiencies, but that is only visible with a direct comparison and could be missed if viewed alone. Burst analysis techniques like BVA and FRET-2CDE provide some of the advanced tools needed to distinguish static states from interconverting states. Again, the small differences are visible in a direct comparison but they are subtle and might not be apparent in isolation.

The conventional histogram analysis methods we applied to the timestamp data used time bins to collect the individual detected photons into an aggregate signal. An aggregate signal is necessary to collect enough FRET signal to overcome the background noise. For the Langevin simulation method, the time bins contain photons with an underlying ensemble of dye–dye distances and efficiencies, but the ensemble becomes averaged over the time of each bin. This is especially true when the underlying dynamics are significantly faster than the bin size. Reducing the size of time bins may reduce the averaging of conformations but also increases the proportion of background noise relative to the smFRET signal. A balance between time bin length and background noise limits how short the time bins can be while containing significant photon counts.

Recent work by Bryan and Pressé^98^ demonstrates how continuous potential energy landscapes can be inferred directly from smFRET data using Bayesian modeling, offering a way to interpret ensemble heterogeneity present in flexible molecules. Our integrated simulation framework complements this study by enabling the generation of synthetic data from known potentials. Specifically, our use of Langevin dynamics to evolve dye–dye distances in predefined potentials offers a validation tool for benchmarking and improvement of methods. Additionally, previous efforts have also focused on generating photon trajectories directly from molecular dynamics simulations, including atomistic modeling of dye fluctuations and accessible volume constraints.^99,100^ Our approach offers a complementary way to simulate dye–dye distance dynamics using Langevin equations enabling flexible and rapid generation of large-scale photon timestamp data sets with full ground truth. Therefore, our method can be used to test and improve analysis tools for smFRET data from conformationally dynamic biomolecules.

It is important to note that the examples of Langevin dynamics provided here (i.e., single-state harmonic potential and two-state bistable potential) are two simple examples provided for illustrative purposes. The methodology provided here can be generalized in a straightforward manner to represent more complex conformational dynamics. For instance, one may use a more complex potential function, *V*(*r*), instead of that used in eqs 2 and 5, containing several minima and maxima. Alternatively, one may use a position-dependent diffusion constant *D*_*L*_(*r*) in eq 1, representing a more realistic conformational diffusion. In more complex cases, one may need to use a multidimensional coordinate space **R**, where *r*(**R**) itself is a function of **R**. In this case potential energy, *V*(**R**), and diffusion coefficient, **D**(**R**) are position-dependent and **R** is evolved according to a general multidimensional overdamped Langevin equation: $R_{i}\left(t+\delta t\right)=R_{i}\left(t\right)-\beta \sum_{j}D_{\mathrm{ij}}\frac{\partial }{\partial R_{j}}V\left(R\right)\delta t+\xi_{i}\left(R\right)$ , where each ξ_*i*_(**R**) is a Gaussian noise related to **D**(**R**).

Among other modifications to the approach is the consideration of dye rotational motion and linker dynamics, that unlike the common assumption, may not be fast enough to be easily integrated out by binning. One may choose to add, in addition to the Langevin dynamics representing the dye–dye distance as in this work, another Langevin dynamics representing the rotational motion of the dyes to more accurately estimate the κ^2^ value as a function of time. Another related modification to consider is the inclusion of linker dynamics. If *r*(*t*) in eq 1 represents the dye–dye distance, *r* itself is a function of the distance between the anchoring points of the labels to the protein (*d*), and two vectors that connect the fluorophores to these anchoring points (**r**_*D*_ and **r**_*A*_). One may use two different sets of 3D overdamped Langevin equations to describe **r**_*D*_(*t*) and **r**_*A*_(*t*) and another one to describe *d*(*t*) and finally calculate *r*(*t*) and κ^2^(*t*) as a function of *d*(*t*), **r**_*D*_(*t*), and **r**_*A*_(*t*). Considerations such as these have been explored in Double Electron–Electron Resonance spectroscopy studies where flexible spin labels have been modeled when interpreting distance distributions.^101–103^ Similarly, studies combing molecular dynamics (MD) simulations and smFRET experiments have modeled linker flexibility and dye orientation to improve the accuracy of distance calculations and κ^2^ estimations.^66,104^

Alternatively, **R** may represent the atomic coordinates of the dye-labeled protein. One may use all-atom MD simulations to generate MD trajectories to calculate dye–dye distance *r*(**R**) and κ^2^(*t*) trajectories. These trajectories can then be incorporated within the PyBroMo software similar to how Langevin simulation data were incorporated in this work. This is an efficient approach to combine MD and Brownian dynamics to provide more accurate freely diffusing smFRET simulation data. As an illustrative example, we have implemented a simplified version of this strategy using the existing MD data for the C-terminal domain of the Albino3 protein (Alb3-Cterm),^28^ an intrinsically disordered protein domain of 138 amino acids. The simulation details are described in ref 28 and include the use of well-tempered metadynamics^105^ of unlabeled Alb3-Cterm in explicit water to generate 16 independent 320 ns long trajectories of Alb3-Cterm. Since the simulations were performed without the dyes, it is not possible to directly measure the dye–dye distance or κ^2^ from these simulations. Therefore, in ref 28, cysteine-attached Alexa488 and Alexa594 dye MD simulations were performed in explicit water and then the generated dye conformers were used to be artificiality attached to the protein at specific positions (C14 and S52). This specific method was developed to allow for attaching dyes to arbitrary locations without the need to rerun simulations for each given position. While this approach may compromise accuracy, it allows for a greater flexibility. Here we used the dye–dye distance trajectories from 16 well-tempered MD simulations (each containing 320 ns of dye-attached Alb3-Cterm protein) to represent 16 independent molecules. For simplicity, we did not calculate κ^2^ (particularly given that the dyes were attached artificially) and used eq 4 for efficiency-distance conversion similar to ref 28 (and similar to the harmonic free energy example above) with *R*_0_ = 57 Å. Since 320 ns is too short for mimicking smFRET data, we arbitrarily changed the time scale of the simulations from 320 ns to 60 s and used a modified version of PyBroMo to simulate 16 freely diffusing molecules with parameters (box size, *D*_*B*_, PSF parameters, maximum emission rate, background rates, and time step) similar to the Langevin dynamics examples above. The threshold for binning was set to 20 counts per bin similar to that used in ref 28 We note that the well-tempered MD simulations allow for much faster transition between different conformational states as compared to unbiased MD; therefore, it is justified to assume the effective time scale of these MD simulations is significantly more than 320 ns. However, the actual conversion factor is not easy to determine. Here, we are using this example for illustrative purposes and do not intend to provide an accurate recipe.

Because the relationship between biased simulation time and physical time is system-dependent and nontrivial to compute, we do not attempt to extract or interpret absolute dynamical time scales from this trajectory. In addition, we note that in this proof-of-concept example, the fluorescent dyes were attached to the protein coordinates in postprocessing rather than being explicitly included during the MD simulation. As a result, the example should not be interpreted as a quantitatively accurate MD–smFRET model. The intent of this illustration—placed in the Discussion for this reason—is only to demonstrate that the Langevin-based framework can accept conformational statistics derived from molecular simulations.

We have shown the results in Figure 7, where we compare both distance distributions and FRET efficiency distributions obtained directly from the MD dye–dye distance trajectories and from the combined MD-PyBroMo approach. As seen in Figure 7, the two are qualitatively similar but there is a shift in both cases. While MD approach takes into account the protein flexibility, the combination with PyBroMo allows for a more realistic FRET efficiency estimation. Also, Figure S5 in the Supporting Information shows experimental freely diffusing smFRET burst data for the same Alb3-Cterm construct, together with a brief discussion of its qualitative correspondence to the simulated distance landscape.

**Figure 7:**
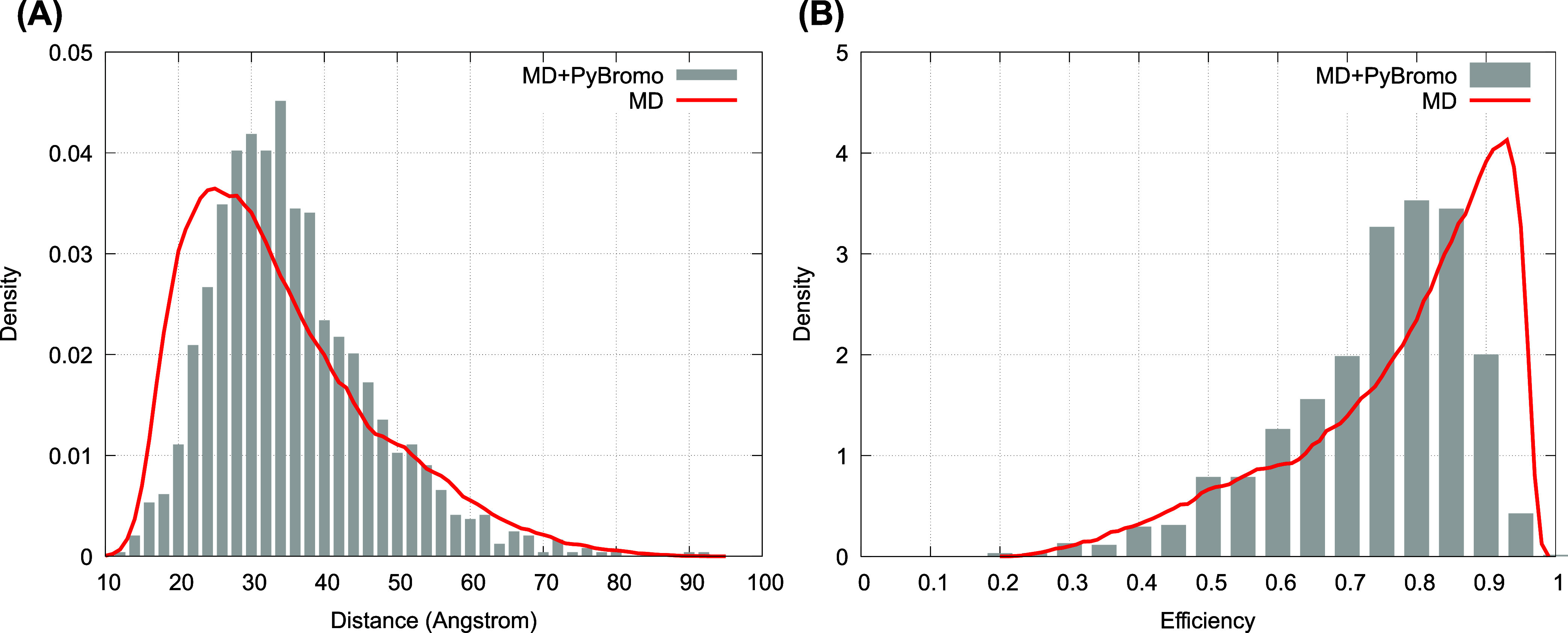
(A) Distance and (B) FRET efficiency distributions from our combined MD-PyBroMo approach for Alb3-Cterm protein (gray) along with the raw MD-based estimates (red). In (A), MD-based distances were binned and normalized directly while the MD+PyBroMo efficiencies were converted to distance before binning and normalization. In (B), MD+PyBroMo efficiencies were binned and normalized directly while the MD-based distances were converted to efficiencies before binning and normalization. Equation 4 and its inverse were used for distance-efficiency conversions.

In using Langevin dynamics to include conformational heterogeneity of the labeled biomolecule, researchers will have the ability to repeatedly generate large amounts of data with a known ground truth of heterogeneous dye–dye distances. Different simulation parameters can easily be changed to generate timestamps and test assumptions based on experimental diffusing smFRET data of flexible molecules with heterogeneous states. New analysis methods beyond the standard time bin methods can then be developed and tested against the simulated data with a known ground truth to assess the effectiveness of such approaches with the ultimate goal of extracting more information from diffusing smFRET experiments of flexible molecules.

## Conclusions

In conclusion, we have shown that the addition of Langevin dynamics to freely diffusing smFRET simulations is capable of generating timestamp data with more realistic heterogeneity of the conformational dynamics of the labeled biomolecule. The purpose of this manuscript was to show how protein conformational dynamics could be added to a typical smFRET data simulator. We compared the Langevin and non-Langevin models to show that in both cases, it is possible to use common smFRET data analysis techniques to produce data consistent with their corresponding ground truth to a reasonable extent. The non-Langevin model is useful as long as the protein conformational dynamics occurs on a submillisecond time scale. Otherwise, for longer time scales the Langevin model will be more practical. While our current model focuses on simulating dye–dye distance fluctuations using overdamped Langevin dynamics, we intentionally excluded other aspects of smFRET experiments to isolate and study the contributions of the labeled biomolecule’s conformational dynamics alone. With this in mind, it is important to note that an explicit model to consider dye rotational/linker dynamics can be added to the proposed simulator. Furthermore, incorporating dye-host interactions, such as steric or electrostatic effects, would increase realism. We have discussed how this can be done along with other directions for the generalization of the approach including the incorporation of the MD as illustrated with an example. The implementation of the Langevin dynamics provides a flexible approach for defining the underlying dynamics of the molecule with full knowledge of the ground truth. Simulated data with known ground truth of realistic heterogeneous dye–dye distances will play an important role in developing new techniques for the analysis of freely diffusing smFRET data for flexible biomolecules.

## Supplementary Material



## Data Availability

Simulation and analysis scripts are available on GitHub Page: https://github.com/bslgroup/PyBroMo

## Notes

Includes additional details of Simulation Software, quantifying error for HMM analysis, changepoint analysis, BVA: single state analysis, FRET-2CDE analysis, and experimental smFRET analysis example. Includes Supporting Figures 1 to 5 (PDF)

## References

[1] Koshland D. E. (1963). Correlation
of structure and function in enzyme action. Science.

[2] Karplus M., Kuriyan J. (2005). Molecular dynamics and protein function. Proc. Natl. Acad. Sci. U.S.A..

[3] Medina E., Latham D. R., Sanabria H. (2021). Unraveling
protein’s structural
dynamics: From configurational dynamics to ensemble switching guides
functional mesoscale assemblies. Curr. Opin.
Struct. Biol..

[4] Agam G., Gebhardt C., Popara M., Mächtel R., Folz J., Ambrose B., Chamachi N., Chung S. Y., Craggs T. D., de Boer M., Grohmann D., Ha T., Hartmann A., Hendrix J., Hirschfeld V., Hübner C. G., Hugel T., Kammerer D., Kang H.-S., Kapanidis A. N., Krainer G., Kramm K., Lemke E. A., Lerner E., Margeat E., Martens K., Michaelis J., Mitra J., Moya Muñoz G.
G., Quast R. B., Robb N. C., Sattler M., Schneider J., Schröder T., Sefer A., Tan P. S., Thurn J., Tinnefeld P., van Noort J., Weiss S., Wendler N., Zijlstra N., Barth A., Seidel C. A., Lamb D. C., Cordes T. (2023). Reliability and accuracy of single-molecule FRET studies
for characterization of structural dynamics and distances in proteins. Nat. Methods.

[5] Xu Y., Havenith M. (2015). Perspective: Watching
low-frequency vibrations of water
in biomolecular recognition by THz spectroscopy. J. Chem. Phys..

[6] Lerner Eitan., Cordes T., Ingargiola A., Alhadid Y., Chung S., Michalet X., Weiss S. (2018). Toward dynamic
structural biology:
Two decades of single-molecule Förster resonance energy transfer. Science.

[7] Schuler B., Hofmann H. (2013). Single-molecule spectroscopy
of protein folding dynamics-
expanding scope and timescales. Curr. Opin.
Struct. Biol..

[8] Perrin J. (1918). La fluorescence. Ann. Phys..

[9] Förster T. (1948). Zwischenmolekulare
Energiewanderung und Fluoreszenz. Ann. Phys..

[10] Ao Y., Grover J. R., Giffford L., Han Y., Zhong G., Katte R., Li W., Bhattacharjee R., Zhang B., Sauve S., Qin W., Ghimire D., Haque M. A., Arthos J., Moradi M., Mothes W., Lemke E. A., Kwong P. D., Melikyan G. B., Lu M. (2024). Bioorthogonal
click labeling of an amber-free HIV-1 provirus for in-virus single
molecule imaging. Cell Chem. Biol..

[11] Stryer L., Haugland R. P. (1967). Energy transfer:
a spectroscopic ruler. Proc. Natl. Acad. Sci.
U.S.A..

[12] Ha T. (2001). Single-molecule
fluorescence resonance energy transfer. Methods.

[13] Lu H. P. (2005). Probing
single-molecule protein conformational dynamics. Acc. Chem. Res..

[14] Roy R., Hohng S., Ha T. (2008). A practical guide to single-molecule
FRET. Nat. Methods.

[15] Sasmal D. K., Pulido L., Kasal S., Huang H. (2016). Single-molecule fluorescence
resonance energy transfer in molecular biology. Nanoscale.

[16] Haas, E. Intrinsically Disordered Protein Analysis; Springer, 2012; pp 467–498. 10.1007/978-1-61779-927-3_28 22760335

[17] Lerner E., Orevi T., Ben Ishay E., Amir D., Haas E. (2014). Kinetics of
fast changing intramolecular distance distributions obtained by combined
analysis of FRET efficiency kinetics and time-resolved FRET equilibrium
measurements. Biophys. J..

[18] Rahamim G., Chemerovski-Glikman M., Rahimipour S., Amir D., Haas E. (2015). Resolution
of Two Sub-Populations of Conformers and Their Individual Dynamics
by Time Resolved Ensemble Level FRET Measurements. PLoS One.

[19] Miller H., Zhou Z., Shepherd J., Wollman A. J., Leake M. C. (2018). Single-molecule
techniques in biophysics: a review of the progress in methods and
applications. Rep. Prog. Phys..

[20] Sharonda
J LeBlanc K. R. W., Kulkarni Prakash. (1996). Prakash Kulkarni Probing the Interaction
between Two Single Molecules: Fluorescence Resonance Energy Transfer
between a Single Donor and a Single Acceptor. Proc. Natl. Acad. Sci. U.S.A..

[21] Ha T., Ting A. Y., Liang J., Caldwell W. B., Deniz A. A., Chemla D. S., Schultz P. G., Weiss S. (1999). Single-molecule fluorescence
spectroscopy of enzyme conformational dynamics and cleavage mechanism. Proc. Natl. Acad. Sci. U.S.A..

[22] Beausang J. F., Zurla C., Manzo C., Dunlap D., Finzi L., Nelson P. C. (2007). DNA looping kinetics
analyzed using diffusive hidden
Markov model. Biophys. J..

[23] Zhuang X., Bartley L. E., Babcock H. P., Russell R., Ha T., Herschlag D., Chu S. (2000). A single-molecule study of RNA catalysis
and folding. Science.

[24] Nierth A., Kobitski A. Y., Nienhaus G. U., Jäschke A. (2010). Anthracene-BODIPY
Dyads as Fluorescent Sensors for Biocatalytic Diels-Alder Reactions. J. Am. Chem. Soc..

[25] Keller B. G., Kobitski A., Jäschke A., Nienhaus G. U., Noé F. (2014). Complex RNA
Folding Kinetics Revealed by Single-Molecule FRET and Hidden Markov
Models. J. Am. Chem. Soc..

[26] Choi U. B., Weninger K. R., Bowen M. E. (2012). Immobilization
of proteins for single-molecule
fluorescence resonance energy transfer measurements of conformation
and dynamics. Methods Mol. Biol..

[27] Schuler B., Eaton W. A. (2008). Protein folding
studied by single-molecule FRET. Curr. Opin.
Struct. Biol..

[28] Baucom D. R., Furr M., Govind Kumar V., Okoto P., Losey J. L., Henry R. L., Moradi M., Kumar T. K. S., Heyes C. D. (2021). Transient
local secondary structure in the intrinsically disordered C-term of
the Albino3 insertase. Biophys. J..

[29] Eggeling C., Berger S., Brand L., Fries J., Schaffer J., Volkmer A., Seidel C. (2001). Data registration
and selective single-molecule
analysis using multi-parameter fluorescence detection. J. Biotechnol..

[30] Rhoades E., Gussakovsky E., Haran G. (2003). Watching proteins fold
one molecule
at a time. Proc. Natl. Acad. Sci. U.S.A..

[31] Cohen A. E., Moerner W. (2008). Controlling Brownian
motion of single protein molecules
and single fluorophores in aqueous buffer. Opt.
Express.

[32] Selvin, P. R. ; Ha, T. Single-Molecule Techniques; Cold Spring Harbor Laboratory Press, 2008.

[33] Santra K., Smith E. A., Song X., Petrich J. W., Bayesian A. (2019). Approach for
Extracting Fluorescence Lifetimes from Sparse Data Sets and Its Significance
for Imaging Experiments. Photochem. Photobiol..

[34] Harris P. D., Lerner E. (2022). Identification and
quantification of within-burst dynamics
in singly labeled single-molecule fluorescence lifetime experiments. Biophys. Rep..

[35] Safar M., Saurabh A., Sarkar B., Fazel M., Ishii K., Tahara T., Sgouralis I., Pressé S. (2022). Single-photon
smFRET. III. Application to pulsed illumination. Biophys. Rep..

[36] Gopich I. V., Szabo A. (2012). Theory of the energy transfer efficiency and fluorescence lifetime
distribution in single-molecule FRET. Proc.
Natl. Acad. Sci. U.S.A..

[37] Sanabria H., Rodnin D., Hemmen K., Peulen T.-O., Felekyan S., Fleissner M. R., Dimura M., Koberling F., Kühnemuth R., Hubbell W., Gohlke H., Seidel C. A. M. (2020). Resolving
dynamics and function of transient states in single enzyme molecules. Nat. Commun..

[38] Götz M., Barth A., Bohr S. S.-R., Börner R., Chen J., Cordes T., Erie D. A., Gebhardt C., Hadzic M. C. A. S., Hamilton G. L., Hatzakis N. S., Hugel T., Kisley L., Lamb D. C., de Lannoy C., Mahn C., Dunukara D., de Ridder D., Sanabria H., Schimpf J., Seidel C. A. M., Sigel R. K. O., Sletfjerding M. B., Thomsen J., Vollmar L., Wanninger S., Weninger K. R., Xu P., Schmid S. (2022). A blind benchmark
of analysis tools to infer kinetic rate constants from single-molecule
FRET trajectories. Nat. Commun..

[39] Farooq S., Hohlbein J. (2015). Camera-based single-molecule FRET
detection with improved
time resolution. Phys. Chem. Chem. Phys..

[40] Chen Y., Shen K., Shan S.-O., Kou S. C. (2016). Analyzing Single-Molecule
Protein Transportation Experiments via Hierarchical Hidden Markov
Models. J. Am. Stat. Assoc..

[41] Okamoto K., Terazima M. (2008). Distribution Analysis
for Single Molecule FRET Measurement. J. Phys.
Chem. B.

[42] McKinney S.
A., Joo C., Ha T. (2006). Analysis of Single-Molecule FRET Trajectories Using
Hidden Markov Modeling. Biophys. J..

[43] Okamoto K., Sako Y. (2012). Variational Bayes analysis
of a photon-based hidden Markov model
for single-molecule FRET trajectories. Biophys.
J..

[44] Pirchi M., Tsukanov R., Khamis R., Tomov T. E., Berger Y., Khara D. C., Volkov H., Haran G., Nir E. (2016). Photon-by-photon
hidden Markov model analysis for microsecond single-molecule FRET
kinetics. J. Phys. Chem. B.

[45] Sgouralis I., Madaan S., Djutanta F., Kha R., Hariadi R. F., Presse S. (2019). A Bayesian Nonparametric Approach
to Single Molecule
Förster Resonance Energy Transfer. J.
Phys. Chem. B.

[46] Saurabh A., Fazel M., Safar M., Sgouralis I., Pressé S. (2023). Single-photon smFRET. I: Theory and conceptual basis. Biophys. Rep..

[47] Jeong C., Cho W.-K., Song K.-M., Cook C., Yoon T.-Y., Ban C., Fishel R., Lee J.-B. (2011). MutS switches
between two fundamentally
distinct clamps during mismatch repair. Nat.
Struct. Mol. Biol..

[48] Lerner E., Barth A., Hendrix J., Ambrose B., Birkedal V., Blanchard S. C., Börner R., Sung Chung H., Cordes T., Craggs T. D., Deniz A. A., Diao J., Fei J., Gonzalez R. L., Gopich I. V., Ha T., Hanke C. A., Haran G., Hatzakis N. S., Hohng S., Hong S.-C., Hugel T., Ingargiola A., Joo C., Kapanidis A. N., Kim H. D., Laurence T., Lee N. K., Lee T.-H., Lemke E. A., Margeat E., Michaelis J., Michalet X., Myong S., Nettels D., Peulen T.-O., Ploetz E., Razvag Y., Robb N. C., Schuler B., Soleimaninejad H., Tang C., Vafabakhsh R., Lamb D. C., Seidel C. A., Weiss S. (2021). FRET-based dynamic
structural biology: Challenges, perspectives and an appeal for open-science
practices. eLife.

[49] Schrimpf W., Barth A., Hendrix J., Lamb D. C. (2018). PAM: A Framework
for Integrated Analysis of Imaging, Single-Molecule, and Ensemble
Fluorescence Data. Biophys. J..

[50] Nettels, B. ; Schuler, D. Fretica; University of Zurich, 2020; available online (https://schuler.bioc.uzh.ch/programs/.

[51] simFCS. Laboratory for Fluorescence Dynamics, 2021; available online (https://www.lfd.uci.edu/globals/).

[52] Gomes G.-N. W., Krzeminski M., Namini A., Martin E. W., Mittag T., Head-Gordon T., Forman-Kay J. D., Gradinaru C. C. (2020). Conformational
ensembles of an intrinsically disordered protein consistent with nmr,
saxs, and single-molecule fret. J. Am. Chem.
Soc..

[53] Kuzmenkina E. V., Heyes C. D., Ulrich Nienhaus G. (2006). Single-molecule FRET Study of Denaturant
Induced Unfolding of RNase H. J. Mol. Biol..

[54] Miller J. J., Mallimadugula U. L., Zimmerman M. I., Stuchell-Brereton M.
D., Soranno A., Bowman G. R. (2024). Accounting for fast vs slow exchange
in single molecule FRET experiments reveals hidden conformational
states. J. Chem. Theory Comput..

[55] Schuler B., Soranno A., Hofmann H., Nettels D. (2016). Single-Molecule FRET
Spectroscopy and the Polymer Physics of Unfolded and Intrinsically
Disordered Proteins. Annu. Rev. Biophys..

[56] LeBlanc S., Kulkarni P., Weninger K. (2018). Single Molecule
FRET: A Powerful
Tool to Study Intrinsically Disordered Proteins. Biomolecules.

[57] Mazal H., Haran G. (2019). Single-molecule FRET methods to study
the dynamics of proteins at
work. Curr. Opin. Biomed. Eng..

[58] Benton M., Furr M., Kumar V. G., Polasa A., Gao F., Heyes C. D., Kumar T. K. S., Moradi M. (2023). cpSRP43 is both highly
flexible and stable: structural insights using a combined experimental
and computational approach. J. Chem. Inf. Model..

[59] Zhang R., Jagessar K. L., Brownd M., Polasa A., Stein R. A., Moradi M., Karakas E., Mchourab H. S. (2024). Conformational
cycle
of a protease-containing ABC transporter in lipid nanodiscs reveals
the mechanism of cargo-protein coupling. Nat.
Commun..

[60] Govind
Kumar V., Polasa A., Agrawal S., Kuamr T. K. S., Moradi M. (2023). Binding affinity estimation from restrained umbrella
sampling simulations. Nat. Comput. Sci..

[61] Govind
Kumar V., Ogden D. S., Isu U. H., Polasa A., Losey J., Moradi M. (2022). Prefusion spike protein conformational
changes are slower in SARS-CoV-2 than in SARS-CoV-1. J. Biol. Chem..

[62] Immadisetty K., Polasa A., Shelton R., Moradi M. (2022). Elucidating the molecular
basis of spontaneous activation in an engineered mechanosensitive
channel. Comput. Struct. Biotechnol. J..

[63] Goolsby C., Losey J., Fakharzadeh A., Xu Y., Düker M.-C., Getmansky Sherman M., Matteson D. S., Moradi M. (2023). Addressing the embeddability
problem in transition rate estimation. J. Phys.
Chem. A.

[64] Sindbert S., Kalinin S., Nguyen H., Kienzler A., Clima L., Bannwarth W., Appel B., Müller S., Seidel C. A. (2011). Accurate Distance Determination of Nucleic Acids via
Förster Resonance Energy Transfer: Implications of Dye Linker
Length and Rigidity. J. Am. Chem. Soc..

[65] Hoefling M., Lima N., Haenni D., Seidel C. A. M., Schuler B., Grubmüller H. (2011). Structural
Heterogeneity and Quantitative FRET Efficiency
Distributions of Polyprolines through a Hybrid Atomistic Simulation
and Monte Carlo Approach. PLoS One.

[66] Deplazes E., Jayatilaka D., Corry B. (2011). Testing the use of molecular dynamics
to simulate fluorophore motions and FRET. Phys.
Chem. Chem. Phys..

[67] Ingargiola A., Laurence T., Boutelle R., Weiss S., Michalet X. (2016). Photon-HDF5:
open data format and computational tools for timestamp-based single-molecule
experiments. Proc. SPIE-Int. Soc. Opt. Eng..

[68] Maruyama G. (1955). Continuous
Markov processes and stochastic equations. Rendiconti
del Circolo Matematico di Palermo.

[69] O’Brien E. P., Morrison G., Brooks B. R., Thirumalai D. (2009). How accurate
are polymer models in the analysis of Förster resonance energy
transfer experiments on proteins?. J. Chem.
Phys..

[70] Hofmann H., Soranno A., Borgia A., Gast K., Nettels D., Schuler B. (2012). Polymer scaling laws of unfolded and intrinsically
disordered proteins quantified with single-molecule spectroscopy. Proc. Natl. Acad. Sci. U.S.A..

[71] Soranno A., Holla A., Dingfelder F., Nettels D., Makarov D. E., Schuler B. (2017). Integrated view of
internal friction in unfolded proteins
from single-molecule FRET, contact quenching, theory, and simulations. Proc. Natl. Acad. Sci. U.S.A..

[72] Milo, R. ; Phillips, R. Cell Biology by the Numbers; CRC Press, 2015.

[73] Nauman J. V., Campbell P. G., Lanni F., Anderson J. L. (2007). Diffusion
of Insulin-Like
Growth Factor-I and Ribonuclease through Fibrin Gels. Biophys. J..

[74] Kuzmenkina E. V., Heyes C. D., Nienhaus G. U. (2006). Single-molecule
FRET Study of Denaturant
Induced Unfolding of RNase H. J. Mol. Biol..

[75] Gopich I. V., Szabo A. (2010). FRET efficiency distributions
of multistate single molecules. J. Phys. Chem.
B.

[76] Berezhkovskii A. M., Szabo A. (2019). Committors,
first-passage times, fluxes, Markov states, milestones,
and all that. J. Chem. Phys..

[77] Schuler B. (2005). Single-molecule
fluorescence spectroscopy of protein folding. ChemPhysChem.

[78] Ingargiola A., Lerner E., Chung S., Weiss S., Michalet X. (2016). FRETBursts:
an open source toolkit for analysis of freely-diffusing single-molecule
FRET. PloS One.

[79] Dahan M., Deniz A. A., Ha T., Chemla D. S., Schultz P. G., Weiss S. (1999). Ratiometric measurement
and identification of single diffusing molecules. Chem. Phys..

[80] Eggeling C., Fries J. R., Brand L., Günther R., Seidel C. A. M. (1998). Monitoring conformational dynamics of a single molecule
by selective fluorescence spectroscopy. Proc.
Natl. Acad. Sci. U.S.A..

[81] Michalet X., Colyer R., Scalia G., Ingargiola A., Lin R., Millaud J., Weiss S., Siegmund O. H., Tremsin A. S., Vallerga J. V. (2013). Development of new photon-counting detectors
for single-molecule fluorescence microscopy. Philos. Trans. R. Soc., B.

[82] Lee N. K., Kapanidis A. N., Wang Y., Michalet X., Mukhopadhyay J., Ebright R. H., Weiss S. (2005). Accurate FRET Measurements within
Single Diffusing Biomolecules Using Alternating-Laser Excitation. Biophys. J..

[83] Deniz A. A., Dahan M., Grunwell J. R., Ha T., Faulhaber A. E., Chemla D. S., Weiss S., Schultz P. G. (1999). Single-pair
fluorescence
resonance energy transfer on freely diffusing molecules: Observation
of Förster distance dependence and subpopulations. Proc. Natl. Acad. Sci. U.S.A..

[84] Nir E., Michalet X., Hamadani K. M., Laurence T. A., Neuhauser D., Kovchegov Y., Weiss S. (2006). Shot-Noise Limited Single-Molecule
FRET Histograms: Comparison between Theory and Experiments. J. Phys. Chem. B.

[85] Dempster A. P., Laird N. M., Rubin D. B. (1977). Maximum Likelihood from Incomplete
Data via the EM Algorithm. J/ R. Soc. Stat.
Soc., Series B.

[86] Benaglia T., Chauveau D., Hunter D. R., Young D. (2009). mixtools: An R Package
for Analyzing Finite Mixture Models. J. Stat.
Software.

[87] R Core Team R: A Language and Environment for Statistical Computing. R Foundation for Statistical Computing: Vienna, Austria, 2020

[88] O’Hagan A., Leonard T. (1976). Bayes estimation
subject to uncertainty about parameter
constraints. Biometrika.

[89] Azzalini, A. ; Capitanio, A. The skew-normal and related families; Cambridge University Press, 2014.

[90] Prates M.
O., Cabral C. R. B., Lachos V. H. (2013). mixsmsn: Fitting Finite Mixture of
Scale Mixture of Skew-Normal Distributions. J. Stat. Software.

[91] Schweiger, M. ; Saurabh, A. ; Pressé, S. A cautious user’s guide in applying HMMs to physical systems. arXiv 2025. 10.1063/5.0284206 PMC12882934 41328966

[92] Torella J. P., Holden S. J., Santoso Y., Hohlbein J., Kapanidis A. N. (2011). Identifying
Molecular Dynamics in Single-Molecule FRET Experiments with Burst
Variance Analysis. Biophys. J..

[93] Tomov T., Tsukanov R., Masoud R., Liber M., Plavner N., Nir E. (2012). Disentangling Subpopulations
in Single-Molecule FRET and ALEX Experiments
with Photon Distribution Analysis. Biophys.
J..

[94] Zucchini, W. ; MacDonald, I. L. ; Langrock, R. Hidden Markov models for time series: an introduction using R; CRC Press, 2017.

[95] Kilic Z., Sgouralis I., Pressé S. (2021). Generalizing HMMs to Continuous Time
for Fast Kinetics: Hidden Markov Jump Processes. Biophys. J..

[96] Altman R. M. (2007). Mixed hidden
Markov models: an extension of the hidden Markov model to the longitudinal
data setting. J. Am. Stat. Assoc..

[97] Sergei, L. ; Ron Weiss, S. ; Grobler, J. ; Cournapeau, D. ; Pedregosa, F. ; Varoquaux, G. ; Mueller, A. ; Thirion, B. ; Nouri, D. ; Louppe, G. ; Vanderplas, J. ; Benediktsson, J. ; Buitinck, L. ; Korobov, M. ; McGibbon, R. ; Lattarini, S. ; Niculae, V. ; csytracy; Gramfort, A. ; Huppenkothen, D. ; Farrow, C. ; Yanenko, A. ; Lee, A. Matthew Danielson hmmlearn/hmmlearn: Hidden Markov Models in Python. with scikit-learn like API. 2016, available online https://github.com/hmmlearn/hmmlearn.

[98] Bryan J. S., Pressé S. (2023). Learning continuous
potentials from
smFRET. Biophys. J..

[99] Hoefling M., Grubmüller H. (2013). In silico
FRET from simulated dye dynamics. Comput. Phys.
Commun..

[100] Steffen F. D., Tomaszowski K. H., Ratzke C., Netz R. R., Hugel T. (2021). FRETraj: Integrating
single-molecule spectroscopy with molecular
dynamics. Bioinformatics.

[101] Polyhach Y., Bordignon E., Jeschke G. (2011). Rotamer libraries of
spin labelled cysteines for protein studies. Phys. Chem. Chem. Phys..

[102] Jeschke G. (2012). DEER distance
measurements on proteins. Annu. Rev. Phys. Chem..

[103] Islam S. M., Stein R. A., Mchaourab H. S., Roux B. (2013). Structural Refinement from Restrained-Ensemble Simulations Based
on EPR/DEER Data: Application to T4 Lysozyme. J. Phys. Chem. B.

[104] Spiegel J. D., Fulle S., Kleinschmidt M., Gohlke H. (2016). Failure of the IDA
in FRET Systems at Close Inter-Dye
Distances Is Moderated by Frequent Low *k*
^2^ Values. J. Phys. Chem. B.

[105] Barducci A., Bussi G., Parrinello M. (2008). Well-tempered
metadynamics: A smoothly converging and tunable free-energy method. Phys. Rev. Lett..

